# Supplementary material for: Heterogeneous migration routes of DNA triplet repeat slip-outs
Source: Biophys Rep (N Y). 2022 Aug 11;2(3):100070. doi: 10.1016/j.bpr.2022.100070 (PMC9586884; doi:10.1016/j.bpr.2022.100070)
Supplement: Document S1. Figures S1–S18 and Tables S1–S8 [file mmc1.pdf]

Biophysical Reports, Volume *2*

## Supplemental information

# Heterogeneous migration routes of DNA triplet repeat slip-outs

**Simona Bianco, Tianyu Hu, Oliver Henrich, and Steven W. Magennis**

## Supporting Material

Oligonucleotide sequences

Supplemental Tables 1-8

Supplemental Figures 1-18

## Oligonucleotide sequences

a) **DNA hairpin**

Donor strand: 5' /Biotin/ TGG CGA CGG CAG CGA GGC TTA GCG GCA AAA AAA AAA AAA AAA AAA AAA AAA AAA AAA AGC CGC **X**

where X = T-Alexa488

Acceptor strand: 5' GCC TCG C**Y**G CCG TCG CCA

where Y = T-Cy5

b) **Static 3WJs with $(CAG)_{10}$ slipouts**

X = C-Cy5 and Y = T-Alexa488

**S-CAG-1**

Top: 5’ AGT GGT CAG ACG CAG CAG CAG CAG CAG CAG CAG CAG CAG **X**AG CTC GGC ACT CGT GAT TTG GTC

Bottom: 5' /Biotin/ GAC CAA ATC ACG AGT GCC GAG CGT C**Y**G ACC ACT

**S-CAG-2**

Top: 5' AGT GGT CAG ACG ATG CAG CAG CAG CAG CAG CAG CAG CAG **X**AG CAG CTC ACT CGT GAT TTG GTC

Bottom: 5' / Biotin/ GAC CAA ATC ACG AGT GAG CAT CGT C**Y**G ACC ACT

**S-CAG-3**

Top: 5' AGT GGT CAG ACG ACT ATG CAG CAG CAG CAG CAG CAG CAG **X**AG CAG CAG CTC CGT GAT TTG GTC

Bottom: 5' /Biotin/ GAC CAA ATC ACG GAG CAT AGT CGT C**Y**G ACC ACT

**S-CAG-4**

Top: 5' AGT GGT CAG ACG ACT GGC ATG CAG CAG CAG CAG CAG CAG **X**AG CAG CAG CAG CGT GAT TTG GTC

Bottom: 5' /Biotin/ GAC CAA ATC ACG CAT GCC AGT CGT C**Y**G ACC ACT

**Fully complementary static 3WJ**

Top: 5' AGT GGT CAG ACG ACT ATG CTG CTG CTG CTG CTG CAG CAG **X**AG CAG CAG CTC CGT GAT TTG GTC

Bottom: 5' /Biotin/ GAC CAA ATC ACG GAG CAT AGT CGT C**Y**G ACC ACT

c) **Mobile 3WJs**

X = C-Cy5 and Y = T-Alexa488

**$(CAG)_{10}$**

Top: 5' AGT GGT CAG ACG CAG CAG CAG CAG CAG CAG CAG CAG CAG **X**AG CAG CAG CAG CGT GAT TTG GTC

**$(CAG)_{40}$**

Top: 5' AGT GGT CAG ACG CAG CAG CAG CAG CAG CAG CAG CAG CAG CAG CAG CAG CAG CAG CAG CAG CAG CAG CAG CAG CAG CAG CAG CAG CAG CAG CAG CAG CAG CAG CAG CAG CAG CAG CAG CAG CAG CAG CAG **X**AG CAG CAG CAG CGT GAT TTG GTC

Bottom: 5’ /Biotin/ GAC CAA ATC ACG CTG CTG CTG CGT C**Y**G ACC ACT

**$(CTG)_{30}$**

Top: 5' AGT GGT CAG ACG CTG CTG CTG CTG CTG CTG CTG CTG CTG CTG CTG CTG CTG CTG CTG CTG CTG CTG CTG CTG CTG CTG CTG CTG CTG CTG CTG CTG CTG **X**TG CTG CTG CTG CGT GAT TTG GTC

Bottom: 5' /Biotin/ GAC CAA ATC ACG CAG CAG CAG CGT C**Y**G ACC ACT

## Supplemental Tables

**Table S1**: FRET states obtained from HaMMy after TDP fitting and ML-BIC optimisation of the mobile $(CAG)_{10}$ stitched trajectory.

| FRET State | 1 | 2 | 3 | 4 | 5 | 6 | 7 | 8 | 9 |
|---|---|---|---|---|---|---|---|---|---|
| 6 | 0.07 | 0.29 | 0.46 | 0.61 | 0.82 | 0.92 | | | |
| 7 | 0.07 | 0.27 | 0.44 | 0.61 | 0.82 | 0.92 | | | |
| 8 | 0.07 | 0.27 | 0.44 | 0.60 | 0.72 | 0.84 | 0.93 | | |
| 9 | 0.06 | 0.16 | 0.31 | 0.45 | 0.59 | 0.73 | 0.84 | 0.93 | |
| 10 | 0.05 | 0.07 | 0.16 | 0.29 | 0.46 | 0.60 | 0.74 | 0.86 | 0.93 |

**Table S2**: FRET states obtained from HaMMy after TDP fitting and ML-BIC optimisation of the mobile $(CTG)_{30}$ stitched trajectory.

| FRET State | 1 | 2 | 3 | 4 | 5 | 6 | 7 | 8 | 9 |
|---|---|---|---|---|---|---|---|---|---|
| 6 | 0.10 | 0.26 | 0.40 | 0.58 | 0.76 | 0.90 | | | |
| 7 | 0.09 | 0.22 | 0.38 | 0.45 | 0.61 | 0.78 | 0.91 | | |
| 8 | 0.09 | 0.21 | 0.32 | 0.39 | 0.55 | 0.70 | 0.83 | 0.91 | |
| 9 | 0.09 | 0.18 | 0.30 | 0.40 | 0.49 | 0.61 | 0.73 | 0.85 | 0.92 |
| 10 | 0.08 | 0.15 | 0.27 | 0.41 | 0.46 | 0.60 | 0.74 | 0.85 | 0.92 |

**Table S3:** BIC values determined across the range of states 2-10 by TDP clustering after HaMMy analysis of the unstitched trajectories of mobile $(CAG)_{10}$, $(CTG)_{30}$ and $(CAG)_{40}$. The most sufficient state configuration ($K_{opt}$) is in italics.

| **$(CAG)_{10}$** | | | | | | | | | |
|---|---|---|---|---|---|---|---|---|---|
| | BIC(2) | BIC(3) | BIC(4) | BIC(5) | BIC(6) | BIC(7) | BIC(8) | BIC(9) | BIC(10) |
| $K = 6$ | 1.702 | 1.1429 | 0.64105 | 0.58144 | *0.40066* | 0.53732 | 0.43453 | 0.48167 | 0.60959 |
| $K = 7$ | 1.6338 | 1.0504 | 0.80262 | 0.70353 | *0.57969* | 0.6092 | 0.64236 | 0.68827 | 0.78373 |
| $K = 8$ | 1.684 | 1.0753 | 0.53509 | 0.59855 | *0.32549* | 0.33816 | 0.33816 | 0.40795 | 0.52494 |
| $K = 9$ | 1.6511 | 1.0877 | 0.59207 | 0.5639 | *0.40379* | 0.41682 | 0.43915 | 0.51133 | 0.6382 |
| $K = 10$ | 1.7314 | 1.1724 | 0.67285 | 0.60734 | 0.51146 | ***0.43501*** | 0.48877 | 0.54963 | 0.65184 |
| **$(CTG)_{30}$** | | | | | | | | | |
| | BIC(2) | BIC(3) | BIC(4) | BIC(5) | BIC(6) | BIC(7) | BIC(8) | BIC(9) | BIC(10) |
| $K = 6$ | 1.6982 | 1.1094 | 0.54029 | 0.26208 | *0.22135* | 0.34056 | 0.53085 | 0.56554 | 0.79665 |
| $K = 7$ | 1.7097 | 1.2589 | 0.73208 | 0.5597 | *0.4988* | 0.52362 | 0.70553 | 0.78155 | 1.0309 |
| $K = 8$ | 1.7231 | 1.1149 | 0.52509 | 0.47601 | *0.30891* | 0.30936 | 0.56456 | 0.61878 | 0.80666 |
| $K = 9$ | 1.6307 | 1.204 | 0.79798 | 0.59009 | *0.5285* | 0.54392 | 0.80136 | 0.78663 | 1.0129 |
| $K = 10$ | 1.6445 | 1.2276 | 0.78701 | *0.5693* | 0.58422 | 0.68499 | 0.80706 | 0.93324 | 1.0816 |
| **$(CAG)_{40}$** | | | | | | | | | |
| | BIC(2) | BIC(3) | BIC(4) | BIC(5) | BIC(6) | BIC(7) | BIC(8) | BIC(9) | BIC(10) |
| $K = 6$ | 1.4934 | 1.2911 | 0.94305 | 0.8062 | *0.72884* | 0.82799 | 0.81475 | 0.85936 | 0.94443 |
| $K = 7$ | 1.455 | 1.2176 | 0.934 | 0.80595 | 0.78686 | *0.78487* | 0.82058 | 0.87962 | 0.8991 |
| $K = 8$ | 1.5122 | 1.2672 | 0.94404 | 0.81272 | *0.74124* | 0.79108 | 0.77801 | 0.88824 | 0.93991 |
| $K = 9$ | 1.4663 | 1.229 | 0.9528 | 0.87264 | *0.75532* | 0.79861 | 0.78273 | 0.91051 | 0.95775 |
| $K = 10$ | 1.5381 | 1.2821 | 0.95305 | 0.8125 | *0.73743* | 0.79946 | 0.806 | 0.90872 | 0.93585 |

**Table S4:** FRET states obtained from HaMMy after TDP fitting and ML-BIC optimisation of the mobile $(CAG)_{10}$ unstitched trajectories.

| FRET State | 1 | 2 | 3 | 4 | 5 | 6 | 7 |
|---|---|---|---|---|---|---|---|
| 6 | 0.08 | 0.26 | 0.43 | 0.62 | 0.79 | 0.93 | |
| 7 | 0.08 | 0.28 | 0.44 | 0.62 | 0.80 | 0.93 | |
| 8 | 0.08 | 0.26 | 0.42 | 0.63 | 0.80 | 0.93 | |
| 9 | 0.07 | 0.25 | 0.41 | 0.62 | 0.80 | 0.93 | |
| 10 | 0.07 | 0.22 | 0.37 | 0.52 | 0.67 | 0.83 | 0.94 |

**Table S5:** FRET states obtained from HaMMy after TDP fitting and ML-BIC optimisation of the mobile $(CTG)_{30}$ unstitched trajectories.

| FRET State | 1 | 2 | 3 | 4 | 5 | 6 |
|---|---|---|---|---|---|---|
| 6 | 0.09 | 0.21 | 0.37 | 0.59 | 0.78 | 0.92 |
| 7 | 0.09 | 0.22 | 0.41 | 0.59 | 0.78 | 0.92 |
| 8 | 0.09 | 0.22 | 0.41 | 0.60 | 0.77 | 0.92 |
| 9 | 0.09 | 0.24 | 0.43 | 0.59 | 0.79 | 0.92 |
| 10 | 0.09 | 0.30 | 0.52 | 0.75 | 0.91 | |

**Table S6:** FRET states obtained from HaMMy after TDP fitting and ML-BIC optimisation of the mobile $(CAG)_{40}$ unstitched trajectories.

| FRET State | 1 | 2 | 3 | 4 | 5 | 6 | 7 |
|---|---|---|---|---|---|---|---|
| 6 | 0.08 | 0.26 | 0.42 | 0.58 | 0.77 | 0.91 | |
| 7 | 0.07 | 0.20 | 0.37 | 0.50 | 0.64 | 0.80 | 0.92 |
| 8 | 0.08 | 0.24 | 0.42 | 0.57 | 0.76 | 0.91 | |
| 9 | 0.08 | 0.24 | 0.42 | 0.58 | 0.77 | 0.91 | |
| 10 | 0.08 | 0.23 | 0.42 | 0.58 | 0.77 | 0.91 | |

**Table S7:** Rate constants ($s^{-1}$) obtained from exponential fitting of the dwell time histograms obtained from the optimised TDP clusters of the unstitched trajectories of static $(CAG)_{10}$ 3WJs (S-CAG-1, S-CAG-2, S-CAG-3 and S-CAG-4), the fully complementary 3WJ and the DNA hairpin. The amplitudes corresponding to the rate constants of the biexponential fittings are provided in the brackets.

| | | | |
|---|---|---|---|
| S-CAG-1<br>Monoexponential | | | |
| FRET states | S1 | S2 | S3 |
| $k_1$ | 4.08 | 4.17 | 2.04 |
| Biexponential | | | |
| $k_1$ ($A_1$ %)<br>$k_2$ ($A_2$ %) | 12.49 (64.26%)<br>1.31 (35.74%) | 5.74 (82.62%)<br>0.67 (17.38%) | 2.9 (70.81%)<br>0.85 (29.19%) |
| S-CAG-2<br>Monoexponential | | | |
| FRET states | S1 | S2 | |
| $k_1$ | 3.72 | 2.71 | |
| Biexponential | | | |
| $k_1$ ($A_1$ %)<br>$k_2$ ($A_2$ %) | 8.5 (72.79%)<br>0.9 (27.21%) | 4.5 (69.49%)<br>1.16 (30.51%) | |
| S-CAG-3<br>Monoexponential | | | |
| FRET states | S1 | S2 | S3 |
| $k_1$ | 0.71 | 10.31 | 18.99 |
| Biexponential | | | |
| $k_1$ ($A_1$ %)<br>$k_2$ ($A_2$ %) | 3.8 (53.84%)<br>0.25 (46.16%) | 27.9 (63.40%)<br>3.48 (36.60%) | 21.34 (89.32%)<br>5.85 (10.68%) |
| S-CAG-4<br>Monoexponential | | | |
| FRET states | S1 | S2 | |
| $k_1$ | 0.26 | 12.68 | |
| Biexponential | | | |
| $k_1$ ($A_1$ %)<br>$k_2$ ($A_2$ %) | 1.82 (82.13%)<br>0.11 (17.87%) | 20.64 (39.00%)<br>1.19 (61.00%) | |
| Fully complementary 3WJ<br>Monoexponential | | | |
| FRET states | S1 | S2 | |
| $k_1$ | 5.73 | 2.56 | |
| Biexponential | | | |
| $k_1$ ($A_1$ %)<br>$k_2$ ($A_2$ %) | 7.16 (79.10%)<br>0.26 (20.90%) | N/A<br>N/A | |
| Hairpin<br>Monoexponential | | | |
| FRET states | S1 | S2 | |
| $k_1$ | 2.46 | 2.16 | |
| Biexponential | | | |
| $k_1$ ($A_1$ %)<br>$k_2$ ($A_2$ %) | 4.18 (45.84%)<br>1.74 (54.16%) | 2.33 (92.62%)<br>0.87(7.38%) | |

**Table S8:** Rate constants ($s^{-1}$) obtained from exponential fitting of the dwell time histograms obtained from the optimised TDP clusters of the unstitched trajectories of mobile $(CAG)_{10}$, $(CTG)_{30}$ and $(CAG)_{40.}$ The amplitudes corresponding to the rate constants of the biexponential fittings are provided in the brackets.

| $(CAG)_{10}$ Monoexponential | | | | | | |
|---|---|---|---|---|---|---|
| FRET states | S1 | S2 | S3 | S4 | S5 | S6 |
| $k_1$ | 2.24 | 1.99 | 6.59 | 5.87 | 1.36 | 1.37 |
| Biexponential | | | | | | |
| $k_1$ ($A_1$ %) | 5.8 (53.71%) | 7.98 (46.72%) | 18.9 (65.29%) | 33.32 (53.15%) | 12.08 (45.11%) | 4.04 (37.04%) |
| $k_2$ ($A_2$ %) | 1.04 (46.29%) | 1.07 (56.28%) | 1.59(34.71%) | 1.97 (46.85%) | 0.85 (54.89%) | 0.92 (62.96%) |
| $(CAG)_{30}$ Monoexponential | | | | | | |
| FRET states | S1 | S2 | S3 | S4 | S5 | S6 |
| $k_1$ | 2.11 | 1.08 | 1.56 | 7.52 | 0.6 | 0.91 |
| Biexponential | | | | | | |
| $k_1$ ($A_1$ %) | 12.2 (52.06%) | 16.46 (27.31%) | 5.93 (54.36%) | 31.98 (65.52%) | 4.57 (27.55%) | 6.7 (27.74%) |
| $k_2$ ($A_2$ %) | 0.99 (47.94%) | 0.87 (72.69%) | 0.59 (45.64%) | 1.28 (34.48%) | 0.44 (72.45%) | 0.7 (72.26%) |
| $(CAG)_{40}$ Monoexponential | | | | | | |
| FRET states | S1 | S2 | S3 | S4 | S5 | S6 |
| $k_1$ | 1.32 | 1.62 | 1.48 | 1.3 | 0.91 | 0.76 |
| Biexponential | | | | | | |
| $k_1$ ($A_1$ %) | 10.48 (50.42%) | 8.49 (39.29%) | 12.94 (35.04%) | 18.99 (19.64%) | 2.84 (39.52%) | 9.38 (23.62%) |
| $k_2$ ($A_2$ %) | 0.72 (49.58%) | 1.03 (60.71%) | 1.06 (64.96%) | 1.14 (80.36%) | 0.57 (60.48%) | 0.65 (79.38%) |

## Supplemental Figures

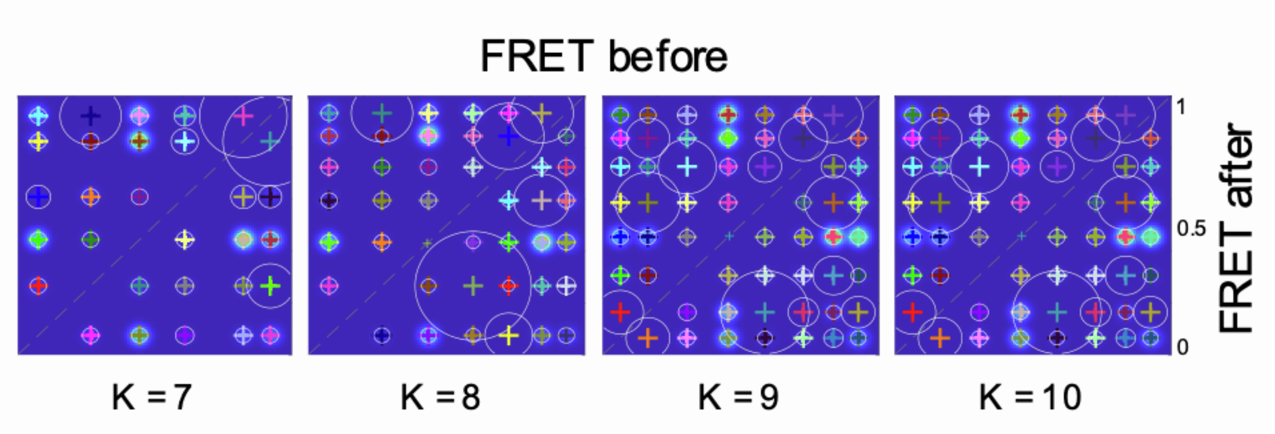


**Figure S1**. TDPs built from the stitched trajectories of mobile $(CAG)_{10}$ for $K = 7, 8, 9, 10$.

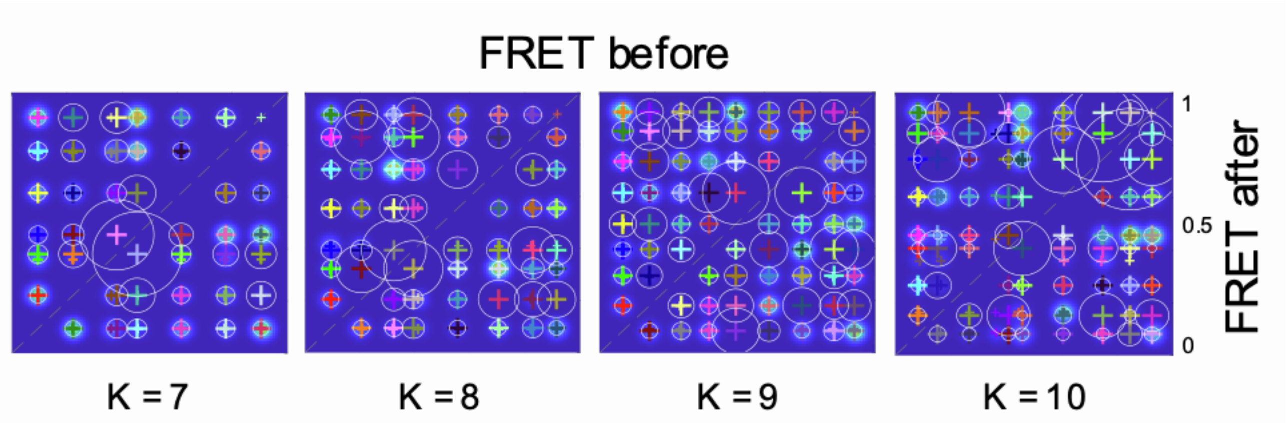


**Figure S2.** TDPs built from the stitched trajectories of mobile $(CTG)_{30}$ for $K = 7, 8, 9, 10$.

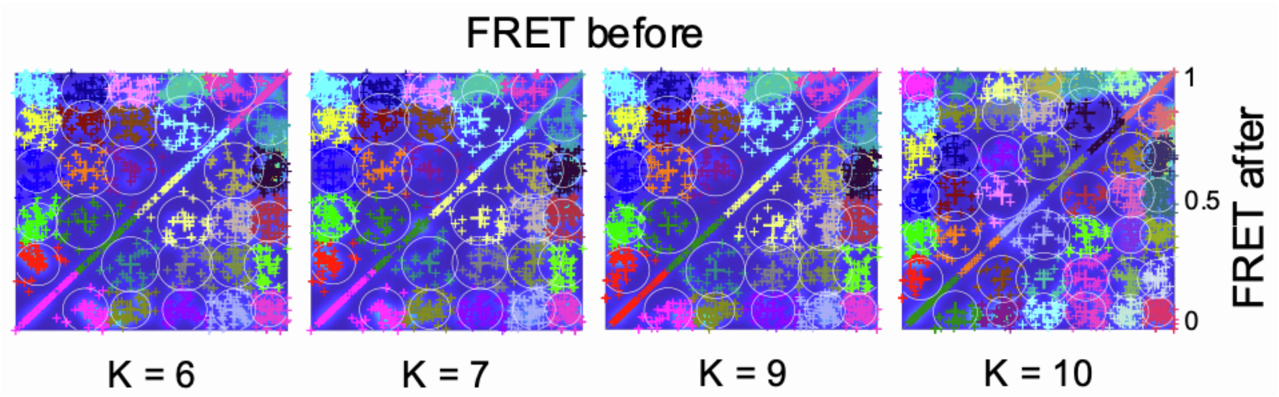


**Figure S3**. TDPs built from the unstitched trajectories of mobile $(CAG)_{10}$ for $K$ = 6, 7, 9, 10.

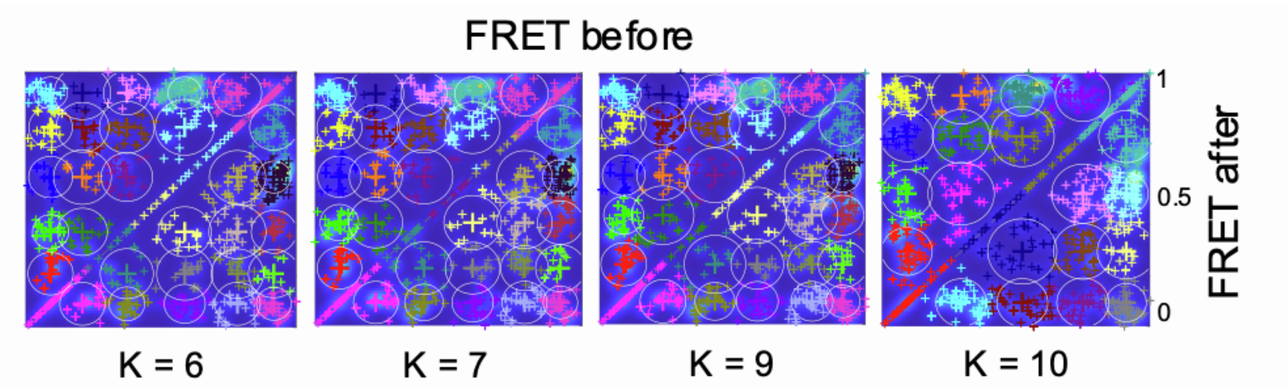


**Figure S4**. TDPs built from the unstitched trajectories of mobile $(CTG)_{30}$ for $K$ = 6, 7, 9, 10.

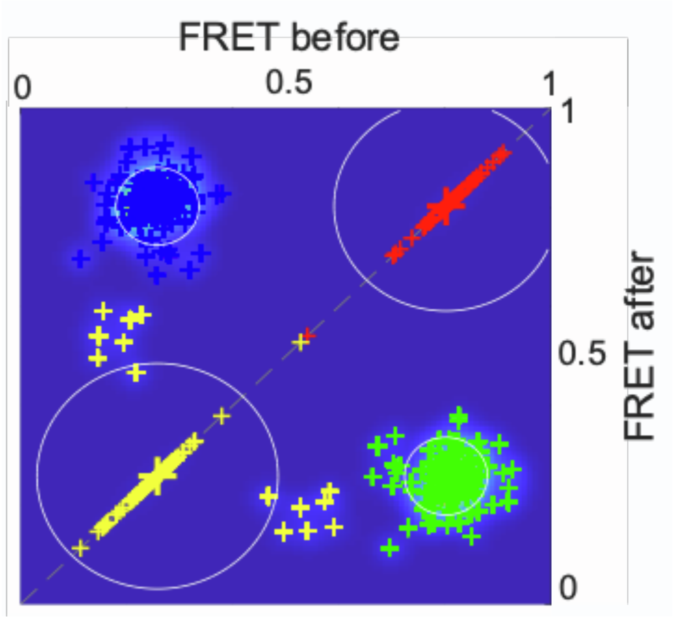


**Figure S5.** TDP from the unstitched trajectories of the DNA hairpin showing two-state dynamics.

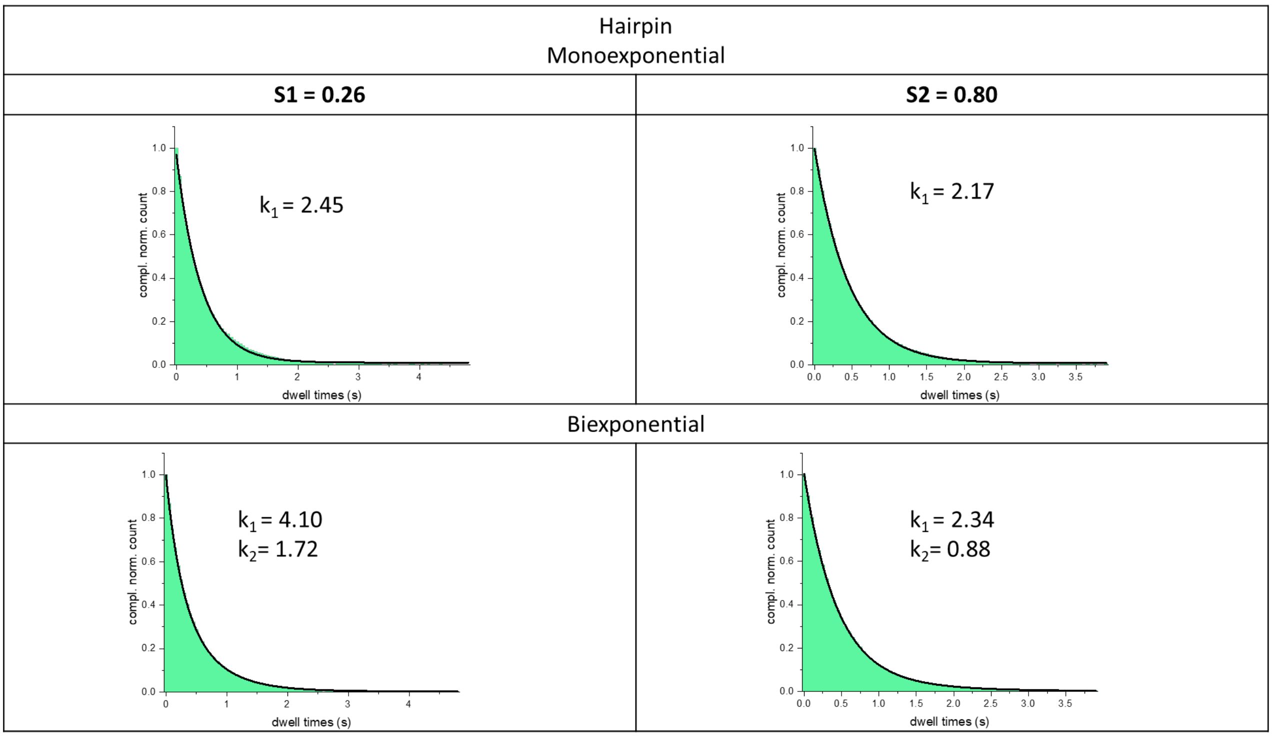


**Figure S6.** Cumulative frequency plots for DNA hairpin showing two-state dynamics.

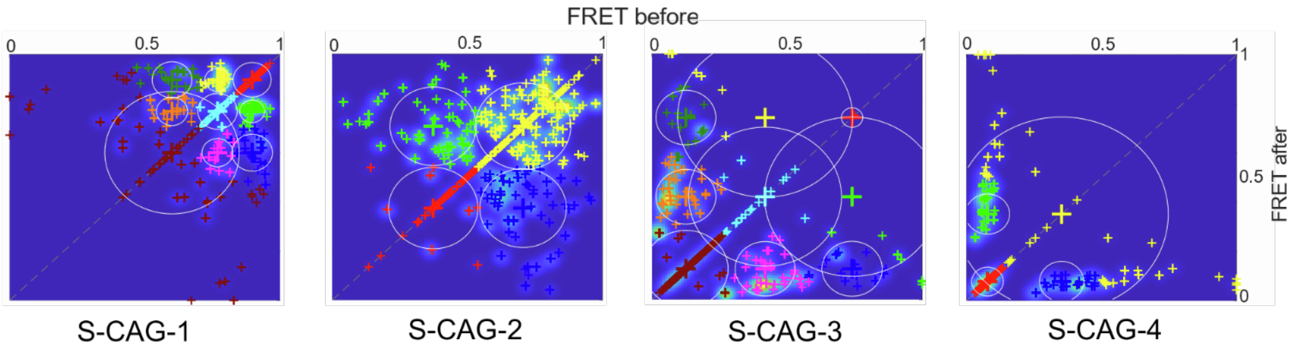


**Figure S7.** TDP from the unstitched trajectories of the static 3WJs.

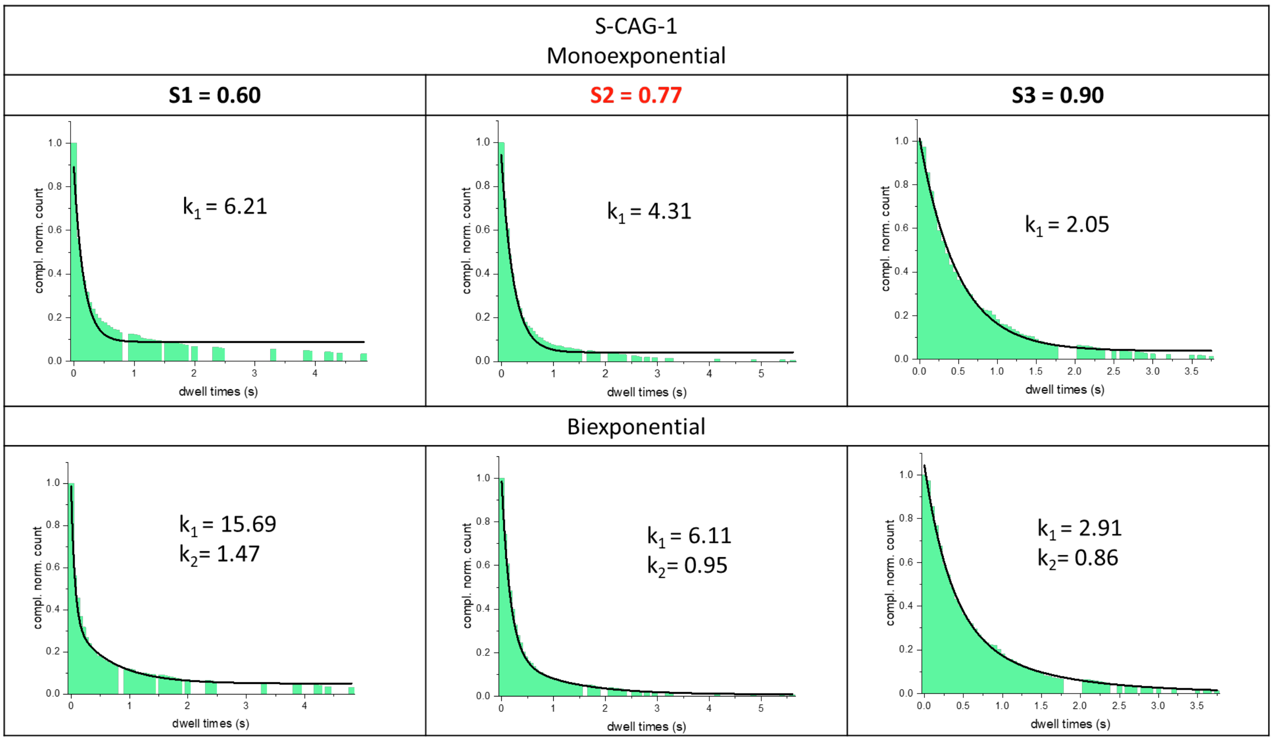


**Figure S8.** Cumulative frequency plots for S-CAG-1, a static $(CAG)_{10}$ 3WJ

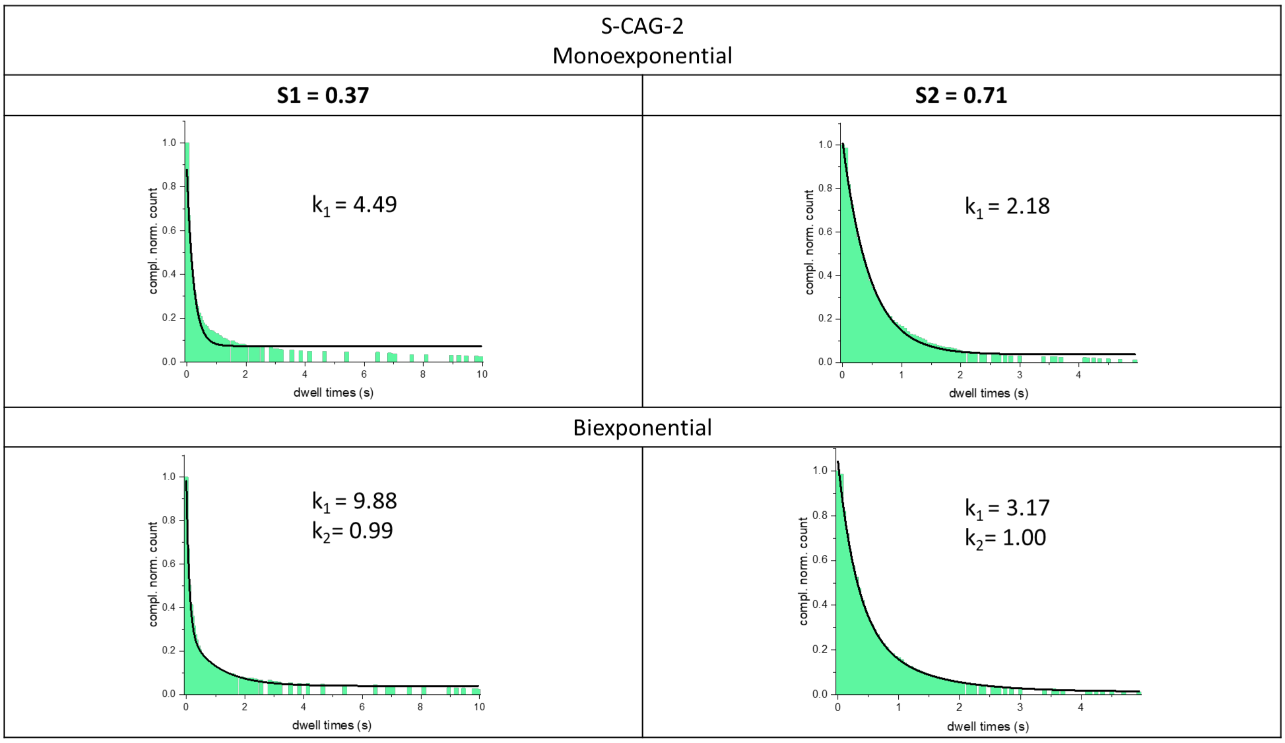


**Figure S9.** Cumulative frequency plots for S-CAG-2, a static $(CAG)_{10}$ 3WJ

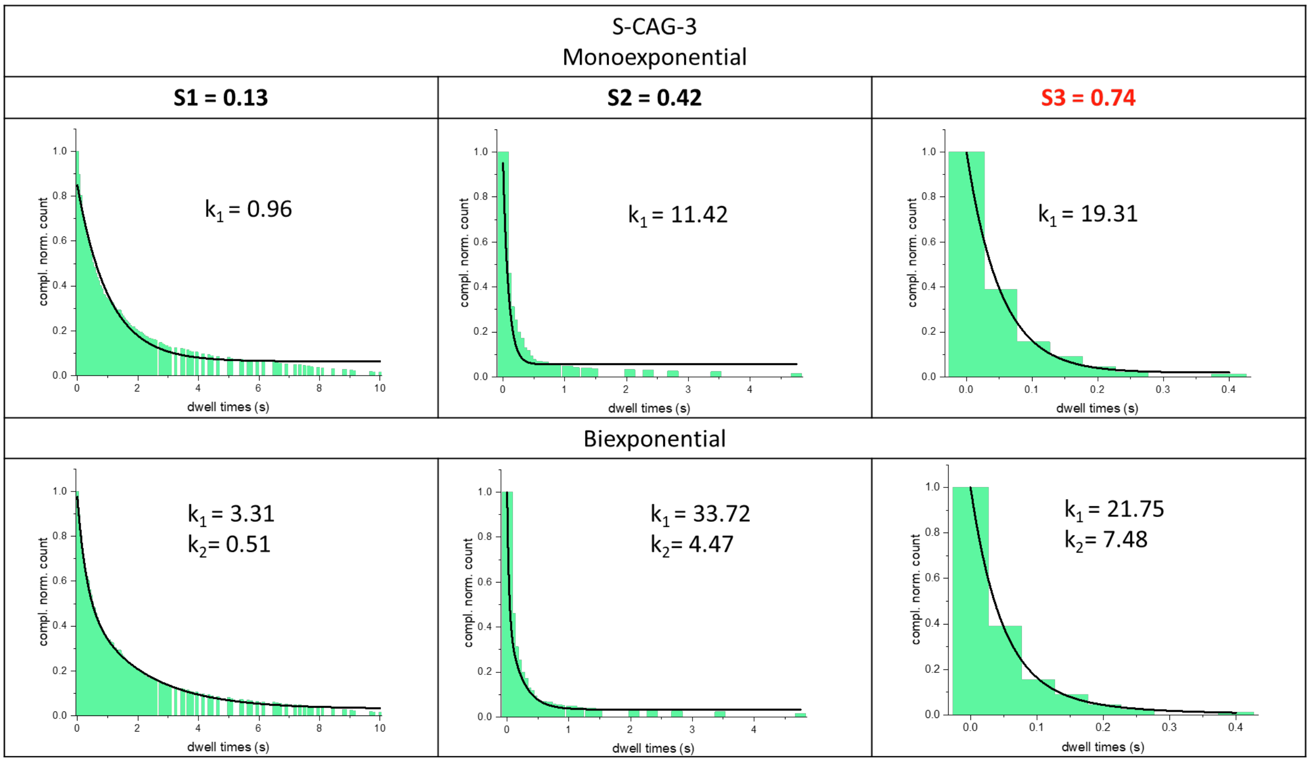


**Figure S10.** Cumulative frequency plots for S-CAG-3, a static $(CAG)_{10}$ 3WJ

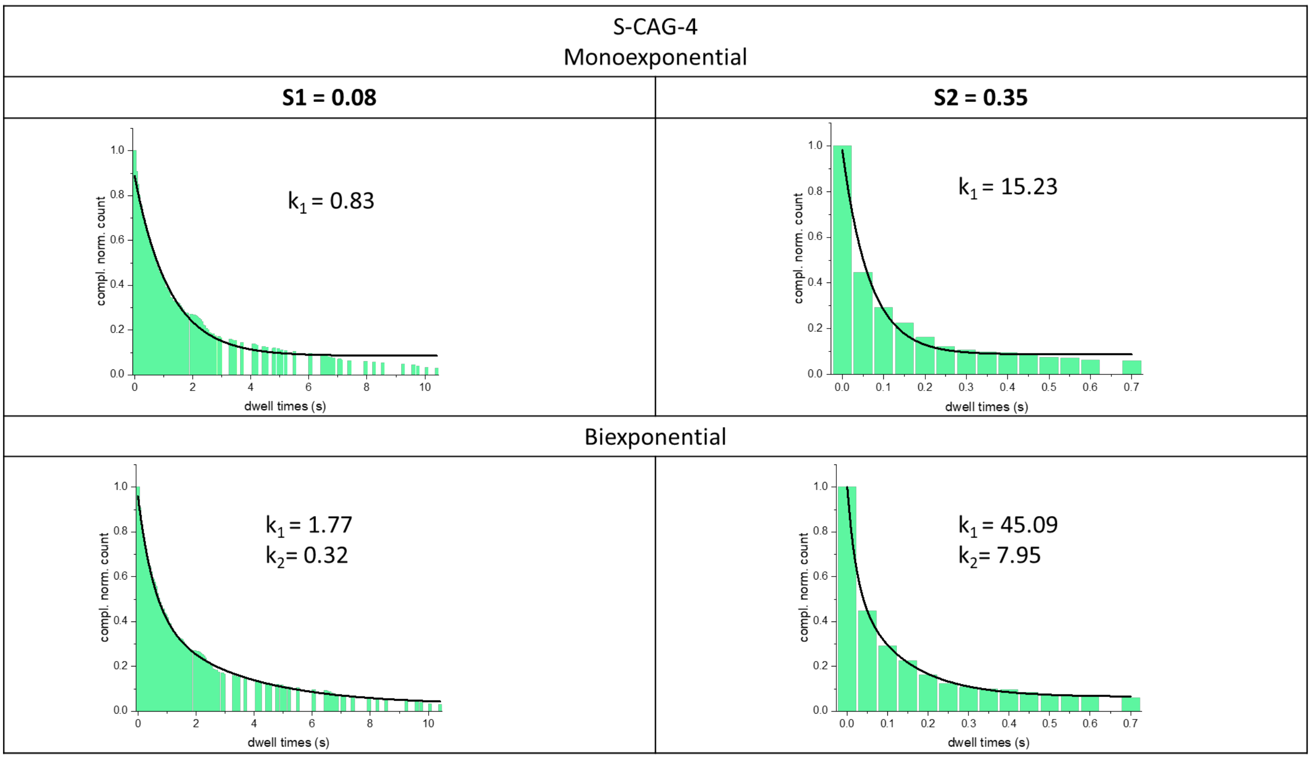


**Figure S11.** Cumulative frequency plots for S-CAG-4, a static $(CAG)_{10}$ 3WJ

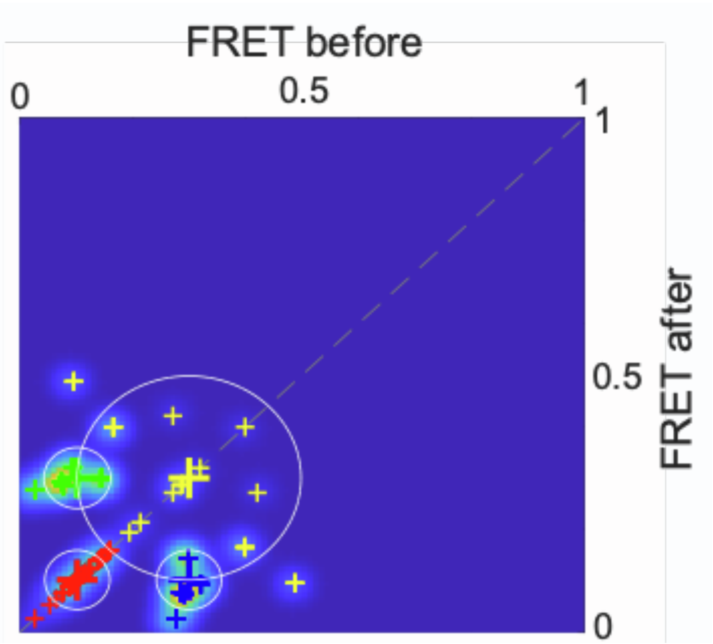


**Figure S12.** TDP from the unstitched trajectories of the fully complementary 3WJ showing two-state dynamics.

| Control: Fully Complementary 3WJ<br>Monoexponential | |
|---|---|
| **S1 = 0.10** | **S2 = 0.30** |
| 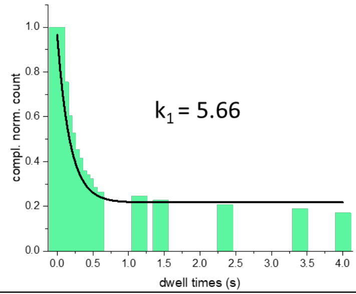  | 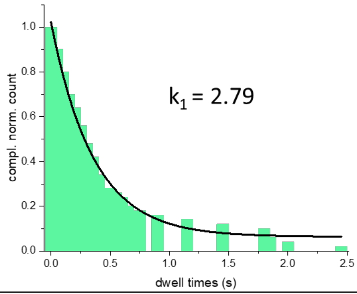  |
| Biexponential | |
| 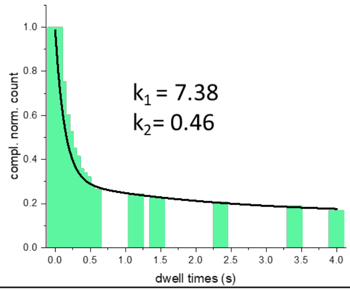  | 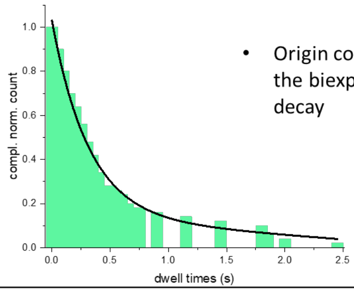  • Origin could not fit the biexponential decay |

**Figure S13**. Cumulative frequency plots for a fully complementary 3WJ

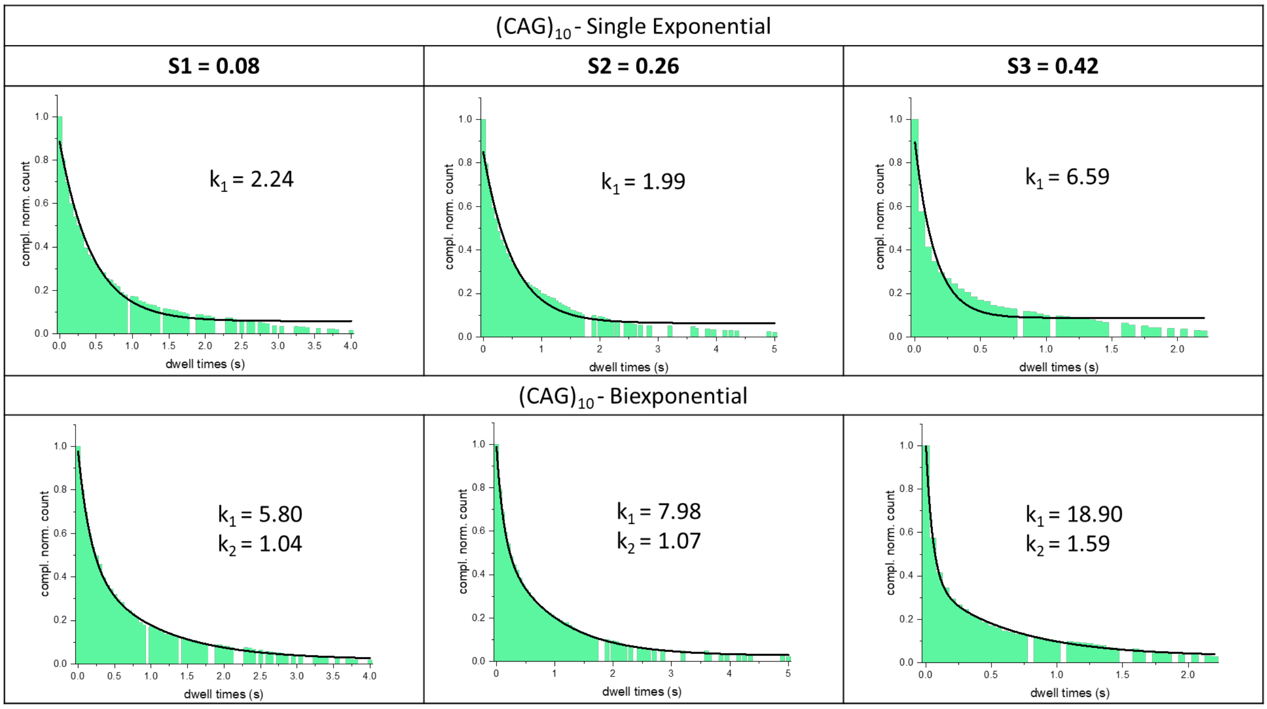


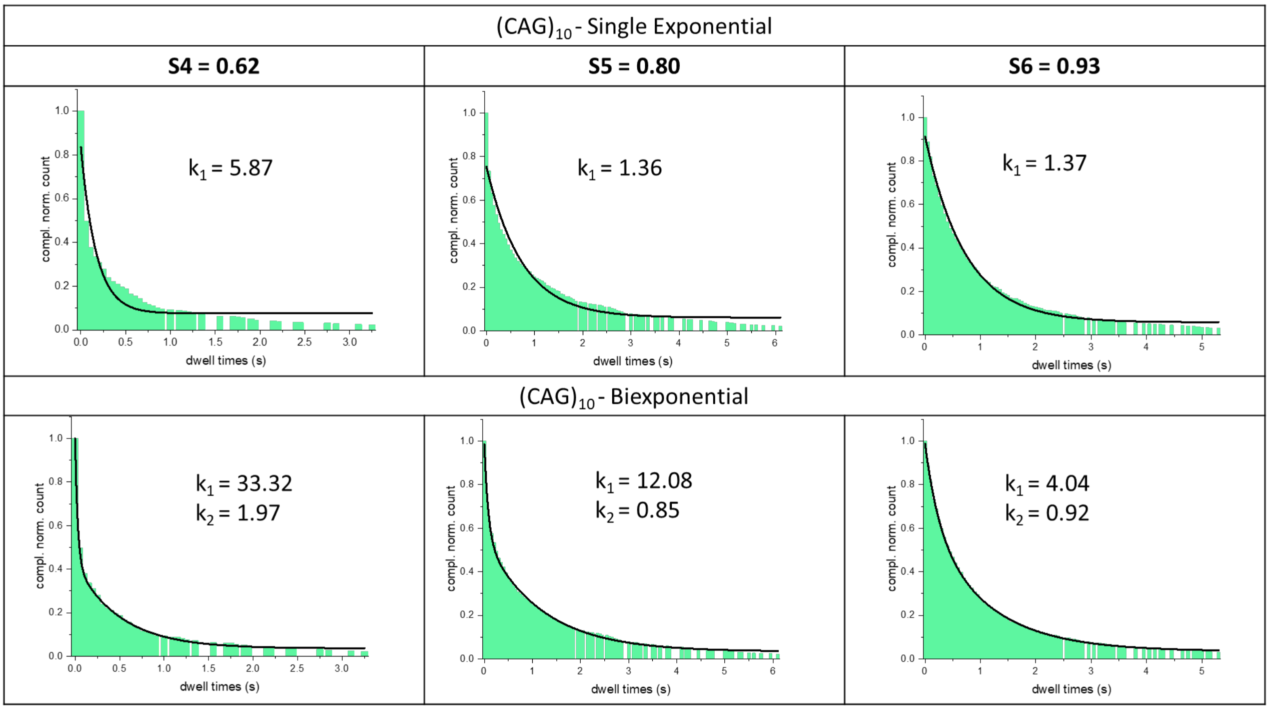


**Figure S14.** Cumulative frequency plots for mobile $(CAG)_{10}$

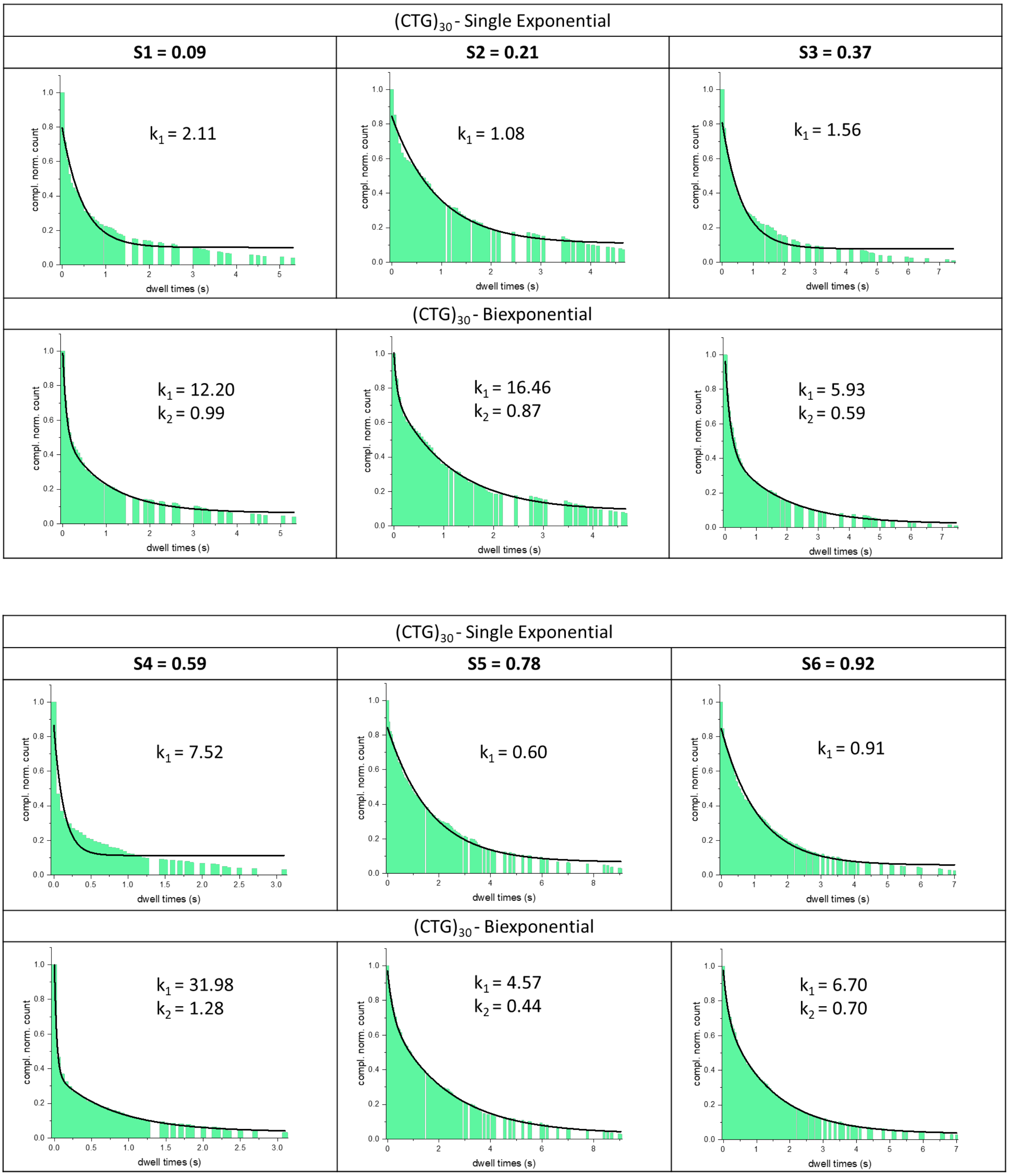


**Figure S15.** Cumulative frequency plots for mobile $(CTG)_{30}$

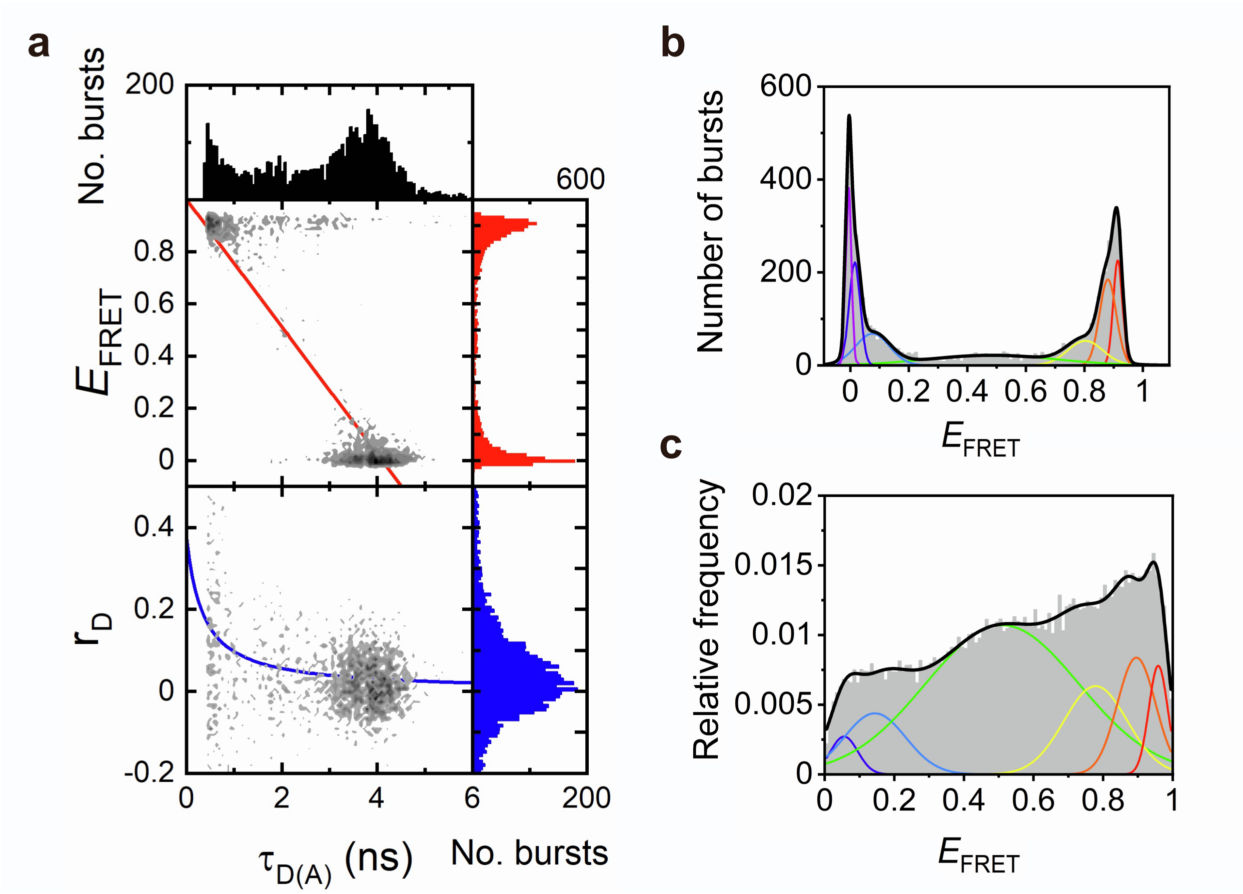


**Figure S16.** SM-FRET of mobile (CAG)$_{40}$ using MFD and TIRF. a) 2D MFD plot shows FRET efficiency ($E_{\text{FRET}}$) or donor anisotropy ($r_D$) vs donor lifetime [$\tau_{D(A)}$]. The overlaid red line is the theoretical FRET relationship $E = 1 - [\tau_{D(A)}/\tau_D]$, where $\tau_D$ = 4.1 ns. The blue overlaid line is the Perrin equation $r_D = r_0/[1 + \tau_{D(A)}/\rho_D]$, with mean rotational correlation time $\rho_D$ = 0.35 ns and fundamental anisotropy $r_0$ = 0.375. The gray scale indicates an increasing number of single-molecule bursts from white to black. The sample was diluted into buffer containing 20 mM Tris, 15 mM NaCl, and 1 mM ascorbic acid at pH 7.5. Before the addition of sample, the buffer was stirred and cleaned with activated charcoal to remove fluorescent impurities. See Hu *et al.* (reference 19 in the main text) for further details of the MFD experiment. b) FRET histogram from MFD fitted to one donor-only state and six FRET states. c) FRET histogram from TIRF fitted to six FRET states using HaMMy.

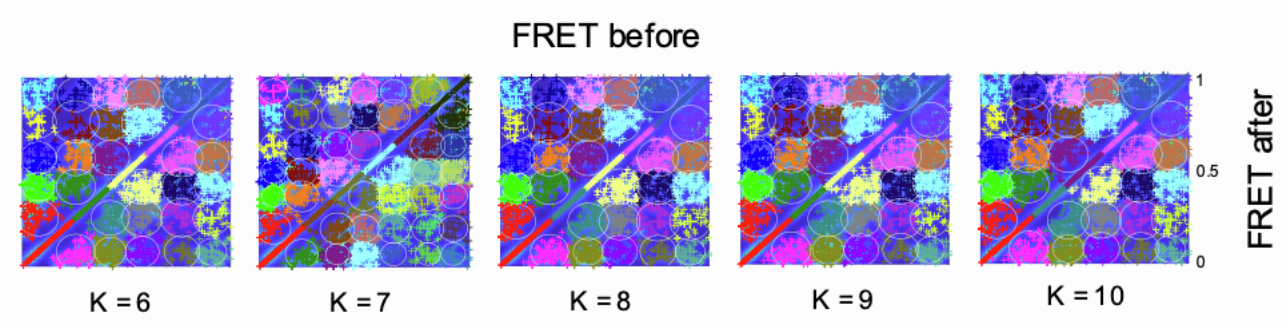


**Figure S17.** TDPs built from the unstitched trajectories of mobile $(CAG)_{40}$ for $K$ = 6, 7, 8, 9, 10.

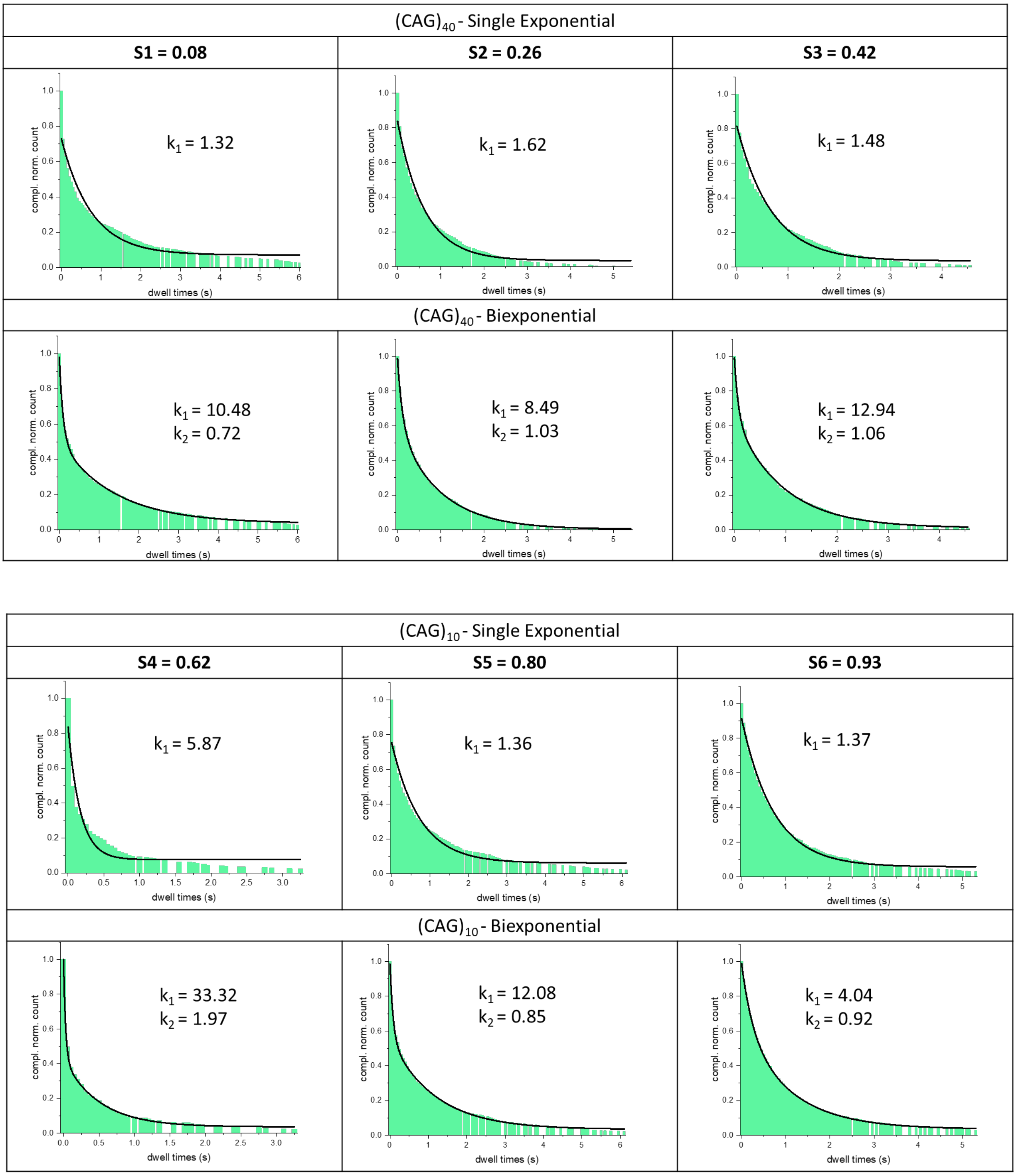


**Figure S18.** Cumulative frequency plots for mobile $(CAG)_{40}$
