# Supplementary material for: Heterogeneous migration routes of DNA triplet repeat slip-outs
Source: Biophys Rep (N Y). 2022 Aug 11;2(3):100070. doi: 10.1016/j.bpr.2022.100070 (PMC9586884; doi:10.1016/j.bpr.2022.100070)
Supplement: Document S2. Article plus supporting material [file mmc2.pdf]

Biophysical Reports

ARTICLE

Heterogeneous migration routes of DNA triplet repeat slip-outs

Simona Bianco,[1] Tianyu Hu,[1] Oliver Henrich,[2,*] and Steven W. Magennis[1,*]
[1]School of Chemistry, University of Glasgow, Glasgow G12 8QQ, UK and [2]Department of Physics, University of Strathclyde, Glasgow G4 0NG, UK

ABSTRACT It is unclear how the length of a repetitive DNA tract determines the onset and progression of repeat expansion diseases, but the dynamics of secondary DNA structures formed by repeat sequences are believed to play an important role. It was recently shown that three-way DNA junctions containing slip-out hairpins of CAG or CTG repeats and contiguous triplet repeats in the adjacent duplex displayed single-molecule FRET (smFRET) dynamics that were ascribed to both local conformational motions and longer-range branch migration. Here we explore these so-called "mobile" slip-out structures through a detailed kinetic analysis of smFRET trajectories and coarse-grained modeling. Despite the apparent structural simplicity, with six FRET states resolvable, most smFRET states displayed biexponential dwell-time distributions, attributed to structural heterogeneity and overlapping FRET states. Coarse-grained modeling for a $(GAC)_{10}$ repeat slip-out included trajectories that corresponded to a complete round of branch migration; the structured free energy landscape between slippage events supports the dynamical complexity observed by smFRET. A hairpin slip-out with 40 CAG repeats, which is above the repeat length required for disease in several triplet repeat disorders, displayed smFRET dwell times that were on average double those of 3WJs with 10 repeats. The rate of secondary-structure rearrangement via branch migration, relative to particular DNA processing pathways, may be an important factor in the expansion of triplet repeat expansion diseases.

WHY IT MATTERS Hairpin DNA structures formed from CAG or CTG repeats are implicated in a number of degenerative diseases. The recent demonstration that such hairpins can migrate along duplex DNA may be important for understanding and treating the disease. Here, the kinetics of migration are characterized in detail using single-molecule microscopy, revealing unexpected dynamic heterogeneity. Coarse-grained modeling supports the migration model, with a free energy barrier and landscape that agrees with the experimental data.

INTRODUCTION

An increasing number of human disorders are attributed to expansions of short tandem repeats. These repeat expansion diseases (REDs) are monogenic, with toxicity mediated at the level of DNA, RNA, or protein.(1) Although expansion is linked to replication,(2,3) growing evidence also supports a major role for replication-independent somatic instability,(4) mediated by mismatch repair (MMR).(5–8) Repeat expansion is also associated with polygenic diseases such as autism spectrum disorder.(9,10) In spite of the variation in RED progression and pathogenesis, there are a number of common features, two of which are particularly relevant here. Firstly, the number of repeats correlates with disease severity and with an earlier age of onset. Secondly, the tandem repeat sequences have an ability to form secondary structures such as hairpins, triplexes, and quadruplexes.(11) These secondary structures are key intermediates in several models for repeat expansion involving processes such as replication, transcription, and repair.(1,6,7)

The most widely studied secondary structure is the hairpin slip-out, which can form when the repeat has partial self-complementarity.(8,12–18) Hu et al. recently reported single-molecule FRET (smFRET) studies of DNA three-way junctions where one arm is a slip-out of trinucleotide repeats (CAG or CTG) of varying length.(19) Expansion of CAG or CTG is the cause of several triplet REDs including Huntington's disease, myotonic dystrophy type 1, and various spinocerebellar

Submitted July 7, 2022, and accepted for publication August 8, 2022.
*Correspondence: oliver.henrich@strath.ac.uk or steven.magennis@glasgow.ac.uk
Editor: Melanie Cocco
https://doi.org/10.1016/j.bpr.2022.100070
© 2022 The Author(s).
This is an open access article under the CC BY license (http://creativecommons.org/licenses/by/4.0/).

ataxias.(1) Hu et al. found that the 3WJs where the triplet repeat sequence extends into the adjacent duplex (so-called mobile 3WJs) can undergo two distinct types of rearrangement: a localized conformational change at the branchpoint (Fig. 1 *a*) and a longer-range interconversion that was assigned to branchpoint migration (Fig. 1 *b*). The latter finding was rather surprising, since it had been previously assumed that long slip-outs were unable to undergo branch migration.(1,13) The report from Hu et al. (19) built on earlier work, which recognized the dynamical nature of small repeat slip-outs (20–22) and isolated hairpins.(23–25) The significance of such processes is not yet established, but they may play a role in disease progression and could be important as therapeutic targets.(19) It is, therefore, important to fully explore the dynamics of these slip-out DNA structures and to establish the mechanism and properties of branch migration.

In this work, we have performed a detailed analysis of the dynamics of smFRET for 3WJs to find the optimal number of FRET states, and to probe the state-to-state kinetics via the dwell time distributions. We also examined the dependence of slip-out size on the dynamics by extending the range to 40 repeats, which is a repeat number that is within the symptomatic range for a number of CAG-associated triplet REDs.(1) To complement the smFRET experiments, we have also employed coarse-grained modeling based on the oxDNA2 model of DNA.(26) (27) Through a combination of experiment and modeling, we have provided compelling additional evidence for the branchpoint migration model that was proposed recently.(19) The coarse-grained modeling confirms the feasibility of migration and gives insight into the complexity of the conformational landscape, which is supported by both the heterogeneity in the smFRET trajectories and the significantly slower kinetics displayed by the 3WJ with a $(CAG)_{40}$ slip-out. We discuss how a competition between branch migration and expansion-prone processing might lead to the characteristic repeat threshold for triplet REDs.

RESULTS

smFRET time-trace analysis workflow

The smFRET trajectories of three mobile DNA 3WJs were analyzed, where mobile refers to those 3WJs that are capable of undergoing branch migration between positional isomers (Fig. 1 *b*).(19) A 3WJ with either 10 or 40 CAG repeats in the slip-out, henceforth referred to as $(CAG)_{10}$ and $(CAG)_{40}$, respectively, and one with 30 CTG repeats in the slip-out, named $(CTG)_{30}$, were studied (see Fig. 2 for all samples studied and Supporting material for details of the oligonucleotides). The $(CAG)_{40}$ sample has not been reported previously. The $(CAG)_{10}$ sample displays kinetics that

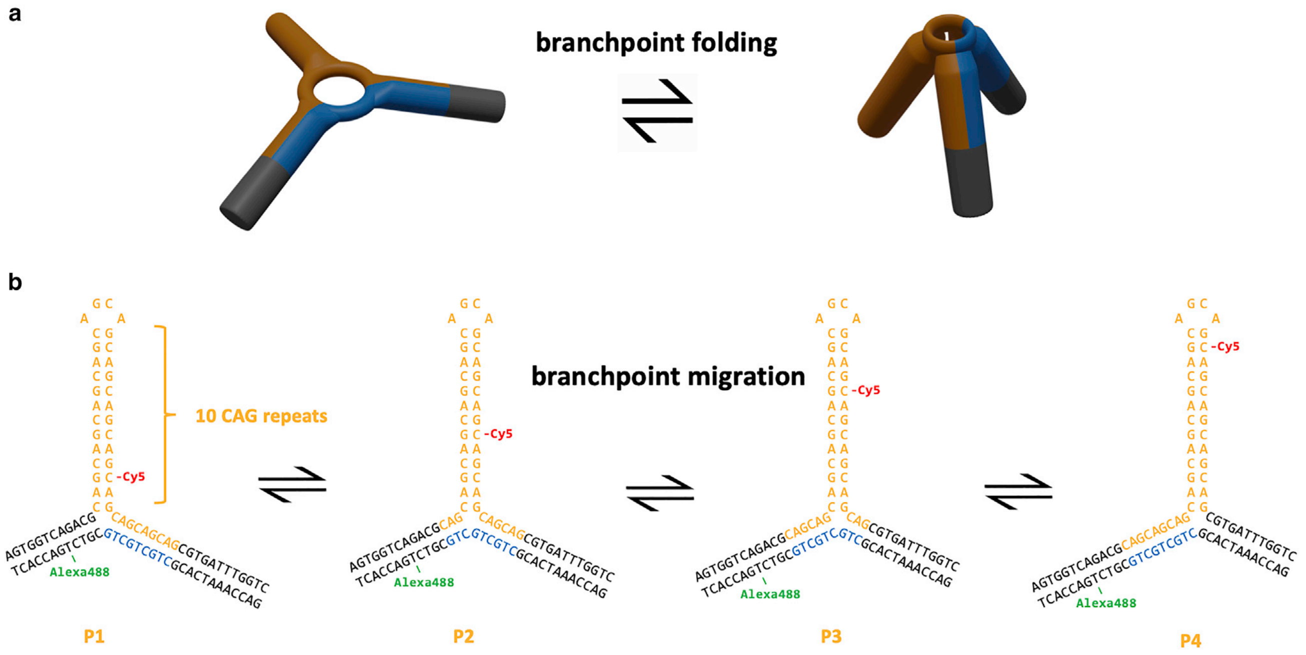

FIGURE 1 Dynamics of three-way DNA junctions incorporating triplet repeat hairpins and contiguous repeats in the adjacent duplex. The dynamics of 3WJs with CAG or CTG repeats in a hairpin were previously described (19) in terms of (*a*) local branchpoint folding and (*b*) interconversion of positional isomers via branch migration (*b*). The picture in (*a*) is adapted from reference (19) (https://creativecommons.org/licenses/by/4.0/).

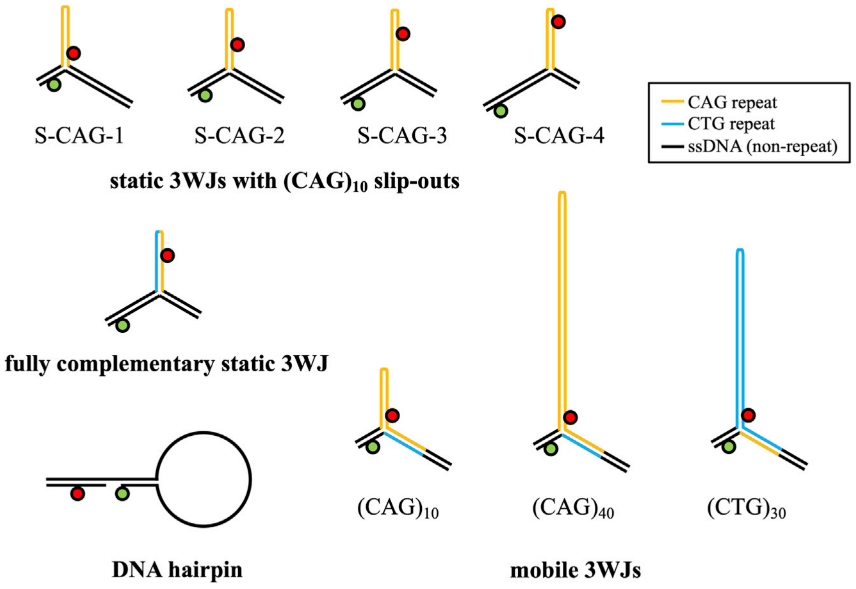

FIGURE 2 Structures studied in this work. See Supporting material for oligonucleotide sequences. The circles represent the donor dye, Alexa488 (green), and the acceptor dye, Cy5 (red).

are representative of all samples with repeat number from 6 to 30.(19) The $(CTG)_{30}$ and $(CAG)_{40}$ samples were chosen because this number of repeats is close to the threshold between healthy and symptomatic ranges for several triplet REDs (e.g., 36 CAG for Huntington's disease). (1)

The overall workflow of the smFRET analysis is illustrated in Fig. 3. First, the time trajectories were fit across a range of states using the HaMMy smFRET analysis software.(28) It is recommended for HaMMy to fit the time traces to $K_{max} + 2$ states, where K_{max} refers to the number of states one expects to observe.(28) Based on the simple model previously proposed, the mobile 3WJs could exist in eight different states (i.e., 2 conformations for each of the four positional isomers).(19) In the previous study, analysis was carried out on the stitched trajectories by constraining HaMMy to $K = 6$. This value was chosen after visual inspection of the fitted time traces and comparison with confocal single-molecule FRET data for freely diffusing 3WJs.(19) In the analysis detailed below, we extended the state range to $K = 6 - 10$. After processing the traces, the

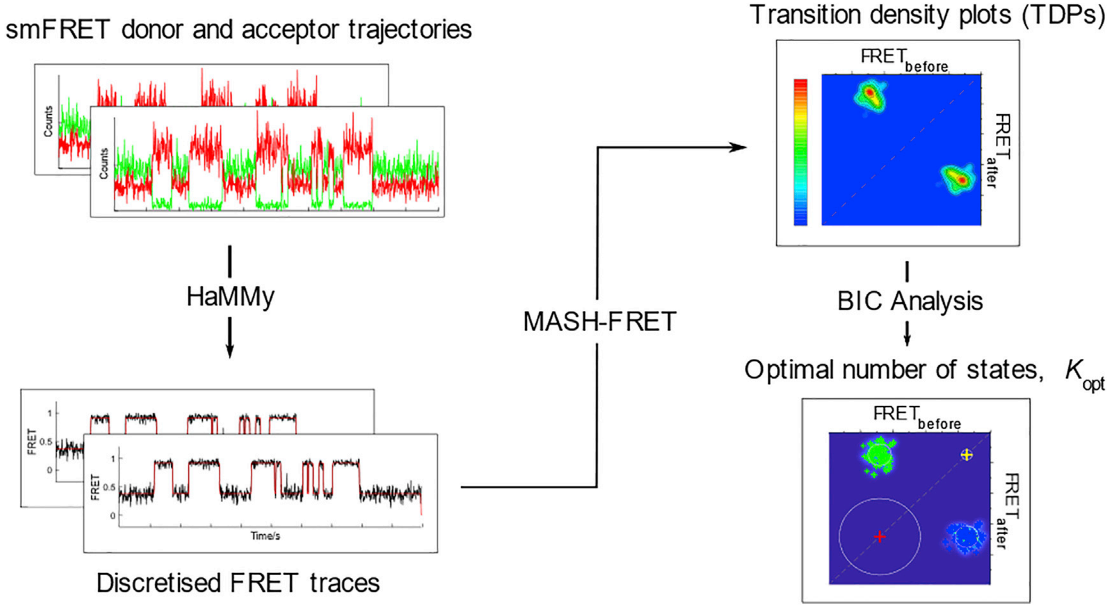

FIGURE 3 smFRET time-trace analysis workflow. smFRET trajectories are first analyzed via HaMMy to obtain discretized FRET traces. The output files are then processed via the MASH-FRET analysis package to produce transition density plots (TDPs) and to calculate the BIC scores to find the optimal number of states K_{opt}.

transitions detected by HaMMy were binned and plotted as transition density plots (TDPs) using the MASH-FRET data analysis package, following the procedure described by Hadzic et al. (29) The TDPs were then fit with two-dimensional Gaussians, and the models were optimized by calculating the Bayesian information criterion (BIC). The goal here was to determine whether a consensus could be reached across the models obtained after analysis, allowing us to determine the optimal number of FRET states, K_{opt}, for the trajectories.

Model selection in the stitched and unstitched smFRET trajectories

In the previous study,(19) the FRET trajectories were stitched into one long trace before analysis by HaMMy. Stitching was performed to avoid the need to carry out postprocessing steps to infer an overall model. However, care must be taken with stitching, since artefactual state-to-state transitions at the boundaries between each trace can be introduced. Moreover, molecular heterogeneity may be obscured during analysis as only one trajectory is considered.(30) In order to examine the effects of stitching, both stitched and unstitched trajectories of the $(CAG)_{10}$ and $(CTG)_{30}$ samples were analyzed according to the workflow above (Fig. 3).

When the stitched trajectories of $(CAG)_{10}$ and $(CTG)_{30}$ were considered, we found that the optimal number of states, K_{opt}, identified by BIC was highly dependent on the number of states K provided during HaMMy analysis (Figs. 4 *a*, S1, and S2, and Tables S1 and S2). In general, the value of K_{opt} was observed to be either the same or one state less than K. This can be understood by considering that the algorithm was constrained to analyze only one long trajectory, so that any molecule-to-molecule variation will result in the fitting of additional states. Since these states are well defined in the TDP, the BIC analysis will also return an overfitted model. In short, particular care must be taken when stitched trajectories are employed for maximum likelihood BIC (ML-BIC) estimation. Specifically, a smaller range of possible states may be more appropriate, based on prior knowledge on the dynamics of the system, as was done previously (19).

There was a marked difference between the ML-BIC fitting for the unstitched trajectories (Figs. 4 *b*, S3, S4, and Tables S3–S6) in comparison to stitched (Fig. 4a). For $(CAG)_{10}$, a consensus across the various results was found for $K_{opt} = 6$, suggesting that 6 states can be reliably resolved from the data (Fig. 4b). Specifically, we note that K_{opt} was found to be different than 6 only for $K = 10$; the requirement to fit seven states could be a result of overfitting during HaMMy analysis. However, even for $K = 10$, the BIC scores for $K_{opt} = 6$ and 7 were close (Table S3), suggesting that the data can be reliably fit to six states. The six states had mean FRET values of 0.08 (0.05), 0.26 (0.06), 0.43 (0.05), 0.62 (0.06), 0.79 (0.05), and 0.93 (0.05); see Materials and methods for details of the errors in parentheses. These values are in agreement with previous findings (19).

For the $(CTG)_{30}$ experiment, a consensus was also achieved for $K_{opt} = 6$ (Figs. 4 *b*). As with $(CAG)_{10}$, a different value was obtained for $K = 10$, where $K_{opt} = 5$. This could be ascribed to states with very similar FRET efficiencies that were clustered into one single state after BIC estimation. Similar to the case of $(CAG)_{10}$, the BIC scores for $K_{opt} = 5$ and 6 were very close (Table S3), again suggesting that six states are consistent with the data. The six states had mean FRET values of 0.09 (0.05), 0.21 (0.06), 0.37 (0.05), 0.59 (0.05), 0.78 (0.06), and 0.92 (0.05). These values are also in good agreement with the ones identified previously.(19)

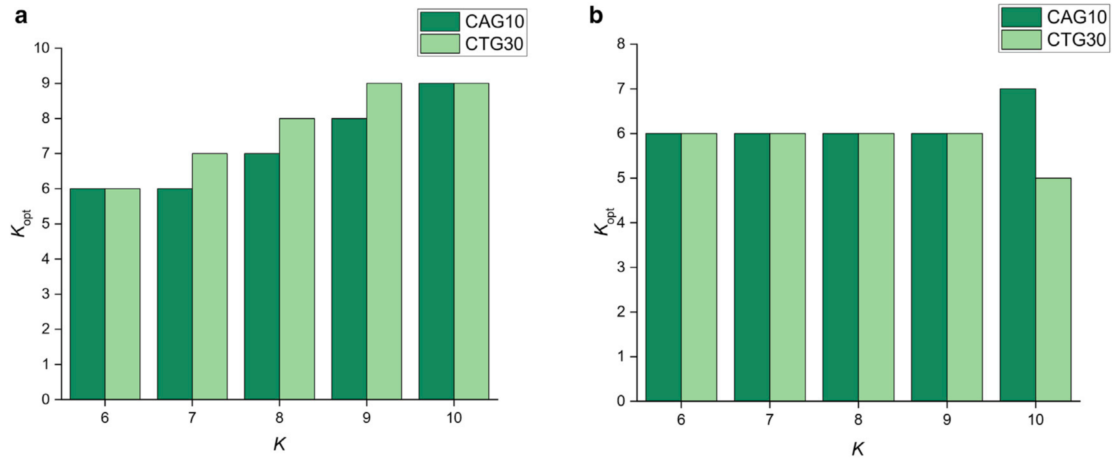

FIGURE 4 Optimization of the number of FRET states. Column plot showing the K_{opt} values obtained by calculating the BIC from the stitched (*a*) and unstitched (*b*) trajectories of $(CAG)_{10}$ and $(CTG)_{30}$ after HaMMy analysis.

Inspection of the TDP clusters of both samples (Figs. 5 and S1–S4) shows that the transition frequencies vary according to the states involved, as supported by the kinetic analysis (see next section). Based on these results, the analysis of individual traces allows assignment of the number of states from complex trajectories in a more unbiased manner, in comparison to those from the stitched trajectories. Specifically, by carrying out model selection via ML-BIC on TDPs, a resistance to overfitting was observed, leading to a consensus across the various discretized state trajectories even for higher values of *K*.

State-to-state smFRET kinetics

In the previous report from Hu et al.,(19) a detailed analysis of the dwell times was not performed. Having established that the data can be described by six FRET states, we next examined the state-to state kinetics of 3WJs with repeat slip-outs. To do this, we use MASH-FRET (29) to generate the normalized cumulative probability distributions (CPDs) of the dwell times for each FRET state. For a Markovian state-to-state process, we would expect these CPDs to fit to a single exponential. To validate the approach, we first analyzed data for a DNA hairpin that was previously shown to obey two-state dynamics.(31) As expected, the TDP (Fig. S5) and dwell times for both states fit to a single exponential (Fig. S6 and Table S7). We then analyzed data for four static 3WJs with $(CAG)_{10}$ slip-outs, a fully complementary 3WJ where the hairpin is composed of $(CTG)_5(CAG)_5$, and mobile 3WJs with $(CAG)_{10}$ or $(CTG)_{30}$ slip-outs; preliminary data had been reported previously for these samples (see Fig. 2 for schematic representations of the structures).(19)

The static 3WJs have almost identical sequences to the mobile $(CAG)_{10}$ 3WJ but lack the contiguous CAG repeats in the duplex immediately adjacent to the slip-out; therefore, they cannot undergo branch migration. Previously it was shown that the static 3WJs could interconvert between two states, which was attributed to a conformational change at the branchpoint, based on earlier work with fully complementary 3WJs.(32,33) We performed the same TDP-BIC analysis as for the mobile 3WJs, with up to four FRET states. For the static 3WJs, we found two differences in comparison with the previous report.(19) Firstly, although two of the static isomers fitted to two states, there was an improvement in the TDP-BIC for two of the isomers by adding a third state (Fig. S7). Secondly, several of the CPDs required a second component to adequately fit the data (Figs. S8–S11). For the fully complementary 3WJ, the time-trace data could be fitted to two FRET states (Fig. S12), both of which exhibited single exponential dwell times; no improvement was found in fitting to a biexponential (Fig. S13 and Table S7). The CPDs for the mobile 3WJs

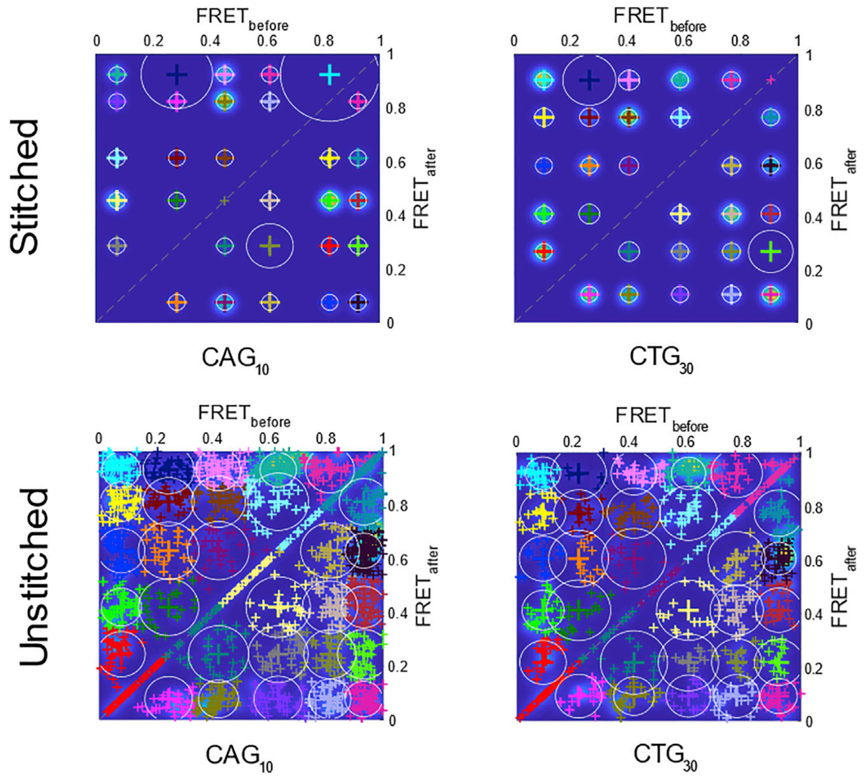

FIGURE 5 Transition density plots for mobile 3WJs. Representative TDPs obtained after clustering the stitched (top) and unstitched (bottom) trajectories of $(CAG)_{10}$ (left) and $(CTG)_{30}$ (right). The TDPs for the trajectories were plotted after running HaMMy with K = 6 and K = 8 for the stitched and unstitched, respectively. The TDP fittings for other values of K are in the Supporting material (Figs. S1–S4). Each colored cluster represents all the transitions assigned from one FRET state ($FRET_{before}$) to a different one ($FRET_{after}$) centered at the $FRET_{before}$ value. The diagonal data arises from traces with no dynamics or low-amplitude artefactual jumps around the same state.

with $(CAG)_{10}$ slip-outs (Fig. S14 and Table S8) and $(CTG)_{30}$ slip-outs (Fig. S15 and Table S8) were qualitatively similar to those of the static samples. The dwell time distributions were best fitted by biexponentials, with the exception of state 5 and state 6, which were monoexponential.

Dependence of FRET dynamics on repeat length: $(CAG)_{10}$ versus $(CAG)_{40}$

Hu et al.(19) presented evidence for a trend toward longer total dwell time with repeat length. In that study, the maximum repeat length was 30. In order to examine the effect of repeat length using the TDP-BIC approach, we also collected data on a mobile 3WJ with 40 CAG repeats (Fig. 2). Apart from the number of repeats, the hairpin is identical to the sample with 10 CAG repeats. Confocal and total internal reflection fluorescence (TIRF) data for freely diffusing or immobilized $(CAG)_{40}$, respectively, recorded in the same way as in the previous study were qualitatively similar to other mobile CAG 3WJs (Fig. S16).(19) As with the mobile $(CAG)_{10}$ and $(CTG)_{30}$ samples discussed earlier, the optimal number of FRET states (for $K = 6 - 10$) was found using MASH-FRET to be 6 (Fig. S17). The six states had mean FRET values of 0.08 (0.05), 0.26 (0.06), 0.42 (0.05), 0.58 (0.05), 0.77 (0.06), and 0.91 (0.05). The only exception was for $K = 7$, where $K_{opt} = 7$; however, the BIC score was very similar between K_{opt} 6 and K_{opt} 7 (Table S3).

As above, the dwell time distributions were examined for each of the six FRET states, using the data obtained for $K_{opt} = 6$. Although some states could be reasonably well fitted to single exponential decays, improvements for all states were found for biexponential fitting (Fig. S18 and Table S8). In order to compare the effect of repeat length on dwell time, the biexponential fits for mobile $(CAG)_{10}$ and $(CAG)_{40}$ were used to produce an average dwell time (inset in Fig. 6) In all cases, particularly for state 3 and state 4, the dwell times for the longer repeat are observed to be longer. The average lifetime, taken from the biexponential fit, which represents the area under the dwell time curve, quantifies this (Fig. 6). The average lifetime increased by a factor ranging from 1.2 to 3.38, with a mean increase (SEM) of 2.0 (0.35). Some transitions will be linked to branchpoint folding rather than migration, and it is possible that migration will have a stronger dependence on the size of the slip-out than folding.

Coarse-grained modeling

Coarse-grained modeling was performed using the oxDNA2 coarse-grained model of DNA (26,27) and its implementation in the Large-scale Atomic/Molecular Massively Parallel Simulator (LAMMPS) (34) via the CG-DNA package (35). As a model, we used a 3WJ with reverse polarity to the $(CAG)_{10}$ that was studied with smFRET (Fig. 1 *b*). Fig. 7 shows representative conformations during a simulated slip-out event. Fig. 7 *a* gives the initial configuration where the nucleotides that hybridize during the slippage event have been marked for clarity in light purple (orange strand 5' – GACGACGAC – 3′) and dark purple (blue strand 5' – GTCGTCGTC – 3′). We also highlight two nucleotides at the top of the hairpin loop (orange strand 5' – CG – 3′) for better visualization of the rearrangements within the hairpin loop. At first, the 3WJ denatures around the branchpoint (Fig. 7 *b*). This results in two very flexible ssDNA regions (Fig. 7 *b*), which quickly form stacks of nucleotides (Fig. 7 *c*), thereby temporarily reducing the free energy again. Unstacking and opening of the hairpin from the stem (Fig. 7 *d*) is followed by the complete opening of the hairpin loop (Fig. 7 *e*) and reclosure from the top (Fig. 7 *f*), whereby the hairpin undergoes a subtle rearrangement that allows it to take in the slack. Then the hairpin stem closes again (Fig. 7 *g*), forming a very stable intermediate state. Then it opens again at the stem (Fig. 7 *h*) and forms again a short ssDNA sequence of stacked nucleotides, visible in Fig. 7 *i* directly in front of the branchpoint. Unstacking (Fig. 7 *j*), opening of the hairpin from the stem (Fig. 7 *k*), complete denaturing of the hairpin loop (Fig. 7 *l*), closure from the top (Fig. 7 *m*), and closure at the stem with re-hybridization occur similarly as before (viz., Fig. 7 *d–i*), completing the branch migration and slippage event in Fig. 7 *n*. It should be noted that Fig. 7 depicts one typical sequence of events. But although the exact sequence differs slightly in individual runs, the shown conformations are generic and representative for all runs we conducted.

We were able to identify several rate-limiting steps: 1) First of all, basepairs in the hairpin stem close to the branchpoint need to denature in order to create partner nucleotides for the slip-out event. 2) The nucleotides in the arm on the right in Fig. 7, which provide the partner nucleotides in the hairpin loop and receiving arm on the left, need to denature and create a bulge of nucleotides in the process that is ready to move up into the hairpin loop. 3) New basepairs need to form in the receiving arm on the left in Fig. 7 through hydrogen bonding with vacant, complementary nucleotides. 4) Finally, the hairpin loop needs to open up and rearrange to take in the ssDNA bulge and reduce stress along the duplex axes. Particularly the last event can take longer as the ssDNA bulge shows a tendency to form an array of stacked nucleotides, which is energetically favorable but prevents the bulge from moving up into the hairpin loop. When this occurs, the 3WJ appears quite distorted and does not resemble a clear

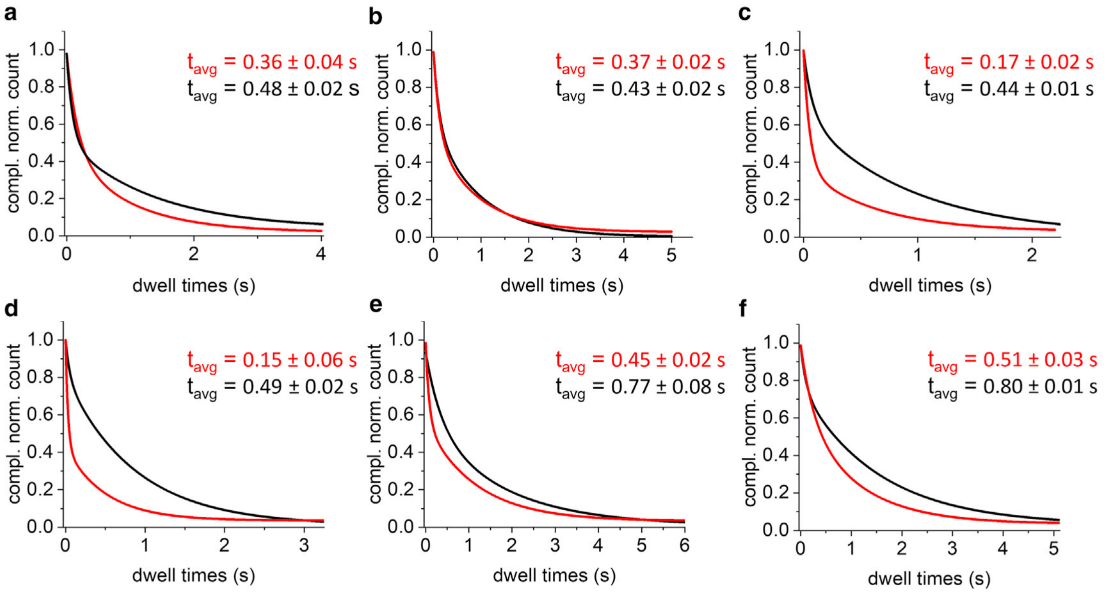

FIGURE 6 Dependence of dwell time on repeat length. Biexponential dwell-time fits for $(CAG)_{10}$ (red) and $(CAG)_{40}$ (black). Panels (*a*)–(*f*) correspond to FRET states 1–6, respectively. The average dwell times from the biexponential fits are shown (inset).

T- or Y-shape. This means that nucleotides that are partners in post-slippage basepairs can be held back at a distance from each other, making the hybridization rather unlikely.

The slippage event seems to occur in two separate, but more or less identical steps: one to two basepairs are formed rapidly in the receiving arm, but further formation is stalled until the aforementioned steps one through four have taken place. When this has happened another two to three basepairs form, bringing the total number of slipped-out nucleotides up to four to five basepairs. Then, steps one through four have to take place again for the remaining nucleotides to undergo slippage, bringing the total number of slipped basepairs up to the final nine in the receiving arm.

It is interesting to analyze the free energy landscape that determines the thermodynamic aspects of the branchpoint migration. Fig. 8 shows the averaged free energy from seven independent runs versus the two collective variables, namely the number of basepairs that need to open up (labeled “denatured bps”) and form (labeled “hybridized bps”) during the slippage; the letters correspond to the snapshots shown in Fig. 7. Both the initial state (a) in the bottom left corner and the final state (n) in the top right corner are characterized by noticeable minima with values around −32 k_BT.

From the heatmap, it becomes obvious there are two regions around (g) and (h), which are relatively easily accessible from the initial state with free energy values around −27 and −23 k_BT, respectively. These regions represent partially slipped states that correspond to the conformations shown in Fig. 7 *g* and *h* with around two to four slipped basepairs. In these states, some new basepairs have already formed in the receiving arm on the left, but the hairpin loop opens only partly at the stem, and the nucleotides in the ssDNA bulge undergo repeated stacking events that block further slippage. However, the state (g) features a hairpin that has taken in the slack and is therefore very stable and relaxed. The state (h) has progressed slightly further in the two reaction coordinates and represents an intermediate conformation before the big slip event. Similar intrastrand interactions have been observed previously in trinucleotide-repeat DNA hairpins,(23) where it was concluded that the repetitive, high mismatch content and self-complementarity gave rise to misfolding and metastable states.

The two basins formed by the initial state (a), including its extension to the states (g) and (h), and the final state (n) representing a full slip-out are separated by another region with free energy values around −17 k_BT, where the intermediate states (k) and (l) are situated. This leads to an effective barrier of around 15 k_BT between the initial state (a) and the final state (n), a value that is comparable with that found in another single-molecule fluorescence study on DNA hairpin dynamics.(36) Using Kramers' reaction rate theory for diffusive barrier crossing, the transition rate between the two positional isomers can be expressed as $k_{f,b} = k_0 \times exp\left(-\frac{\Delta G^{\ddagger}}{k_BT}\right)$

with k_f and k_b as forward and backward rate, respectively, $\Delta G^{\ddagger}$ as the height of the free energy barrier between the two positional isomers, and k_0 as pre-exponential factor. Note we assume here that forward and backward rate are the same, which is justified as

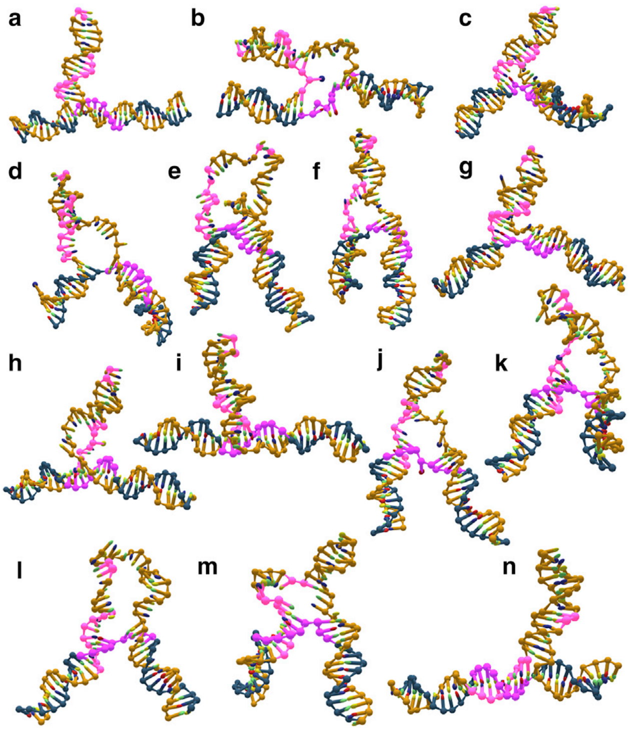

FIGURE 7 Branchpoint migration of the mobile $(GAC)_{10}$ 3WJ using coarse-grained modeling. The pictures show a typical sequence of representative conformational changes during the slippage event: (*a*) initial state, (*b*) providing arm opens, (*c*) stacked bulge, (*d*) hairpin stem opens, (*e*) hairpin loop opens, (*f*) hairpin loop closes, (*g*) hairpin stem closes, (*h*) hairpin stem opens, (*i*) stacked bulge, (*j*) unstacked bulge, (*k*) hairpin stem opens, (*l*) hairpin loop opens, (*m*) hairpin loop closes, and (*n*) final state. The nucleotides in light and dark purple mark the positions of the nine complementary basepairs after slippage (*n*) and the two nucleotides that are situated at the top of the hairpin loop before the slippage (*a*) (see main text for sequence and explanation).

the free energy values in the respective minima related to the two positional isomers in Fig. 8 are virtually identical. Although the exact pre-exponential factor is a piori not known, it is plausible to assume values of the order $k_0 \approx O(10^6)s^{-1}$ as these have been typically found in studies of hairpin dynamics.(36,37) Together with our value of the free energy barrier, this leads to characteristic transition rates of $O(1)s^{-1}$, which are very compatible with our experimental observations. It should be noted that we opted here for the simplest bias that allowed us to observe full slippage events between the initial and final state. A more detailed study and statistical analysis of the slippage events with different initial configurations and longer hairpin loops and different triplet repeat sequences will be conducted in the future.

DISCUSSION

We recently showed that mobile 3WJs with hairpins formed from CTG or CAG repeats exhibit two distinct types of dynamics, involving two-state rearrangement at the local branchpoint and interconversion of positional isomers.(19) In this work we have examined these 3WJ dynamics in detail using smFRET and coarse-grained modeling.

A drawback of smFRET is that the signals are inherently noisy, which has led to the development of specialized methods of analysis.(30) One of the most popular approaches is hidden Markov Modeling (HMM), which is well-suited to analyzing noisy signal trajectories.(38) HMM formulations seek to find the hidden variables within observed time series and identify the most probable path through them.(39) As a result, they allow identification of FRET states that may be hidden by noise in the smFRET signal.(30) Dedicated HMM analysis software for smFRET data has been freely available for over 15 years,(28) and this continues to be an active area of research.(40–48) Previously, Hu et al.(19) employed the software HaMMy developed by McKinney et al.(28) to identify the number of FRET states for DNA 3WJs containing trinucleotide repeats.(19) To estimate the best fitting path for a given signal trajectory, HaMMy uses a maximum likelihood (ML) approach, which aims to find the parameters that maximize the probability of observing a given set of data. McKinney et al.(28) also showed how to use the BIC(49) from the discretized HaMMy traces to infer the most optimal number of states from the HaMMy data by selecting the model that returns the lowest value of the BIC. BIC estimation further improves on direct ML inference for model selection, as it introduces a penalty term for complexity. By avoiding model overfitting, BIC inference can be employed as a powerful tool to obtain the simplest model that best fits the data.(50)

We analyzed individual unstitched trajectories via ML-BIC on TDPs using the MASH-FRET package, which demonstrated a resistance to overfitting and provided a consensus about the number of FRET states. This was not the case for the stitched trajectory, suggesting that particular care needs to be taken when analyzing such complex datasets by this method. Nevertheless, the overall conclusion of the previous work in terms of the conformational and migrational dynamics holds. Rather than finding additional states with the improved time-trace analysis approach employed here, we have resolved the same number of states.

By comparing the FRET states for the static and mobile $(CAG)_{10}$ samples, we can see that all of the static FRET states are present in the time-trace data for the mobile 3WJs. In other words, the mobile 3WJs can interconvert between all four positional isomers. Our simple model of two-state conformational dynamics for each of four positional isomers would produce up to eight FRET states. We therefore might expect one

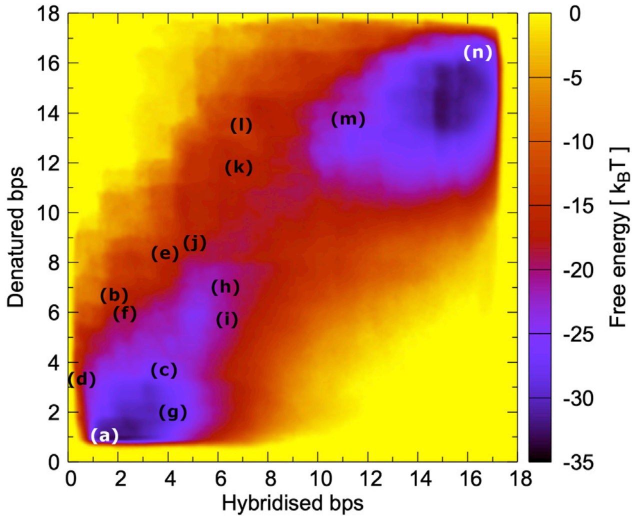

FIGURE 8 Free energy landscape during slippage of a mobile $(GAC)_{10}$ 3WJ. The letters correspond to the snapshots shown in Fig. 7. The initial state (*a*) is situated at a low number of hybridized and denatured basepairs in the bottom left, whereas the final state (*n*) is at the top right. Both states are separated by a free energy barrier, but an area of more accessible conformations exists between the two and constitutes an intermediate, partially slipped-out state (*g*). The result represents the average of seven independent runs.

or two of the FRET states to be triply or doubly degenerate, respectively. However, heterogeneity seems to be the norm, rather than the exception, and it is also evident in the static 3WJ samples. Although, hidden smFRET states have been documented previously, our system (with six degenerate FRET states) is more complex than most systems that have been reported to date.(44–47) As a result of the heterogeneity in FRET states, we are unable to unambiguously assign the individual FRET states to particular 3WJ structures. However, it is clear that almost all transitions are possible. This means that, within the timescale of the experiment, there must be transitions from one positional isomer to its neighbor, but also as far away as the next nearest neighbor.

To support the smFRET experiments, we also adopted a coarse-grained method to study 3WJ dynamics. We studied a 3WJ with a $(GAC)_{10}$ slip-out, using oxDNA2 in combination with the well-tempered metadynamics method.(51) The oxDNA2 model applies a top-down approach to coarse-grained modeling and represents each nucleotide as a single rigid body with effective bonded and pair interactions between the nucleotides. oxDNA2 is able to describe the thermodynamics of duplex formation very accurately and provides a good average representation of the structure of DNA with major and minor groove and the mechanics of both single- and double-stranded DNA and its assemblies. These thermodynamic and structural aspects are very important for investigating structures such as 3WJs, where the conformations deviate significantly from the canonical B-DNA form.

The coarse-grained modeling indicates that a simple bulge-loop model is not appropriate for the hairpins studied here, which are long enough to form stable hairpins. Instead, more complex, metastable, secondary structures prevail, which are formed by intermediate states between the two positional isomers and introduce the requirement of coordinated structural rearrangements for slippage events to occur. The source of both the branch migration and the dynamic heterogeneity is likely to be the local branchpoint and hairpin structure. It is worth noting that dynamic heterogeneity has been reported for the related Holliday junction,(52) where it was attributed to Mg^{2+}-induced folding; the various topologies revealed by modeling here (Fig. 7) are consistent with this. Importantly, this dynamic heterogeneity is absent from both a simple hairpin made from ssDNA and a fully complementary 3WJ hairpin. In other words, the key to the heterogeneity appears to be the mismatches in the hairpin and near the branchpoint. These mismatches presumably allow more conformational flexibility, in agreement with ensemble NMR experiments of static 3WJs.(53) This flexibility could facilitate the branch migration in the mobile 3WJs.(19) For longer slip-outs, additional heterogeneity could result from various multiloop configurations or even tertiary structures.(54)

The possibility of repeat hairpins migrating was discussed previously in the context of spontaneously formed slip-out hairpins, though there was no experimental evidence at that time.(55,56) For very short repeat slip-outs, which are essentially unpaired loops,(19) it has also been proposed that branch migration occurs via soliton-like movement of small bulge loops (Fig. 9 *a*).(20–22) Related to this, isolated hairpins formed from longer repeat sequences have been shown to undergo dynamic rearrangements, which have been attributed to small loop movement.(24,25) These isolated hairpins could form via a nick in a single strand of duplex DNA, a potential source of instability.(25) Although the dynamics observed in the 3WJs is clearly related to those of the small bulge loops and isolated hairpins above, placing a longer repeat hairpin in the context of duplex DNA is rather different, as was noted previously.(55)

Based on earlier work showing that a single base mismatch is a major barrier to Holliday junction branch migration,(57) it was assumed that 3WJ branch migration does not to occur.(1) However, up to four contiguous internal mismatches were required to significantly slow strand displacement by an invading single strand in dsDNA.(57) Indeed, a single strand of DNA with a run of three mismatched bases was capable of displacing an otherwise complementary strand from a 3WJ, albeit

at a reduced rate.(58) Therefore, mismatches per se are not a major obstacle. We have also presented evidence that the 3WJ dynamics increase with repeat length. Since stable hairpins can migrate (Fig. 9 *b*), this opens up the possibility that the likelihood of harmful expansion might relate to the relative rate of branch migration and expansion-prone processing. In support of this, it is known that the length of the pure CAG repeat length in Huntington's disease is a better predictor for the age of disease onset than the length of the translated polyglutamine (from CAG with CAA interrupts).(59) The model of branch migration presented earlier(19) and supported here relies on the self-complementarity of the repeat, and it would be impeded by such interrupts.

Migration could involve movement of the slip-out to a less or more error-prone site, or it could result in merging of hairpin loops on opposite strands to recover the duplex (Fig. 9 *b*). It is possible that similar migrational dynamics also occur for other secondary structures (e.g., triplexes or quadruplexes). At some length of repeat, there could be a tipping point where secondary structure relocation or reversal slows and expansion-prone processing takes over. With this model, the repeat length threshold for a specific RED would be set by the relative rate of secondary-structure dynamics and the specific downstream cause of pathogenesis.

Although this is plausible, evidence from replication-independent somatic expansion suggests that DNA expands and contracts by only a few repeats at a time, which points toward smaller bulges as the key secondary structure that are incorrectly processed.(4,5,60–62) However, if it is a smaller loop that is processed, why is there such a strong dependence on repeat length, and why does there appear to be such an important role for longer slip-outs? Small loops are essentially fast moving bulges and would be expected to migrate relatively freely.(20–22) Although longer repeats would require small loops on opposite strands to traverse greater distances before merging, this does not seem to explain the rather dramatic dependence on repeat length. Furthermore, CAG and CTG repeats certainly form stable hairpins, and these have been implicated in various expansion mechanisms.(2,3,7,63,64) Hairpins have also been observed in vivo, where somatic expansion correlated with levels of slipped DNA.(65)

It is possible that there is more than one mechanism for expansion, depending on the length of the slip-out, the repeat sequence, the sequence context, the tissue, etc. However, it is also feasible that there is a single model that might reconcile the different viewpoints. Kaplan et al. postulated that a universal model for trinucleotide expansion diseases might exist, which would involve an increasing probability of somatic expansion as a function of repeat length.(66) More recently, a dependence of expansion on repeat length has been demonstrated.(67) We propose here a mechanism of somatic expansion that involves both large and small loops (Fig. 9 *c*). Although a short repeat sequence may preferentially form small loops, these might be expected to migrate quickly and recover the stable duplex (Fig. 9 *a*). As the number of repeats increases, it might be expected that larger and larger hairpins can form (Fig. 9 *b*). Although not processed by MMR, longer hairpins are recognized by the mismatch repair machinery.(68) We speculate that the large slip-outs may have a role in blocking the migration and merging of the small loops. As the larger hairpins grow, they might become increasingly stable and less prone to migrating and re-annealing, giving time for the cellular machinery to process the small loops, ultimately leading to expansion.

MATERIALS AND METHODS

Sample preparation

Oligonucleotides were synthesized and labeled by Purimex (Grebenstein, Germany) and IBA (Göttingen, Germany). Oligonucleotides were purified by double HPLC and PAGE. The NHS-esters of Alexa488 (5′/6′ mixed isomer, Invitrogen) or Cy5 (GE-Healthcare), or Atto647N were attached via a 5-C_6-aminoallyl-dC or 5-C_6-aminoallyl-dT. The sequences of oligonucleotides are shown in the Supporting material.

Annealing of samples for branched DNA was carried out in buffer (20 mM Tris (Sigma-Aldrich) and 50 mM NaCl (Fluka), pH 7.5) with the ratio of donor strand to acceptor strands at 1:2. Samples were heated to 90°C in a water bath and left to cool down slowly overnight. For TIRF measurements (see below), the buffer contained 20 mM Tris-HCl, 10 mM NaCl (pH 7.8) with 6% glucose (w/w), 2 mg/mL glucose oxidase, 0.08 mg/mL glucose catalase, and 1 mM Trolox to reduce the rate of blinking and photobleaching of the dyes. The measurement buffer contained 1 mM $MgCl_2$ (Fluka). All measurements were recorded at 20°C ± 1°C.

Objective-type total internal reflection fluorescence microscopy data

TIRF experiments were performed, and text files containing the TIRF trajectories were generated as reported previously.(19) Briefly, the TIRF signals were recorded with an exposure time of 50 ms and were processed using TwoTone (v3.1).(69) Photobleaching removal and generation of donor and acceptor intensity time traces was achieved using a custom MATLAB script.

Evaluation of the number of states

Several hundred single-molecule FRET trajectories for each sample were analyzed to evaluate the optimal number of states, K_{opt}. In the previous study these traces were stitched. Here we used the same traces either stitched or unstitched. Trace processing was performed using the HMM software HaMMy (v4.0).(28) In order to test the robustness of the analysis method, the FRET efficiency trajectories were fit across a range of states, specifically from 6 to 10. HaMMy required a reasonably long time to analyze the stitched trajectories, especially when the value of K exceeded 7 or 8. For example, when

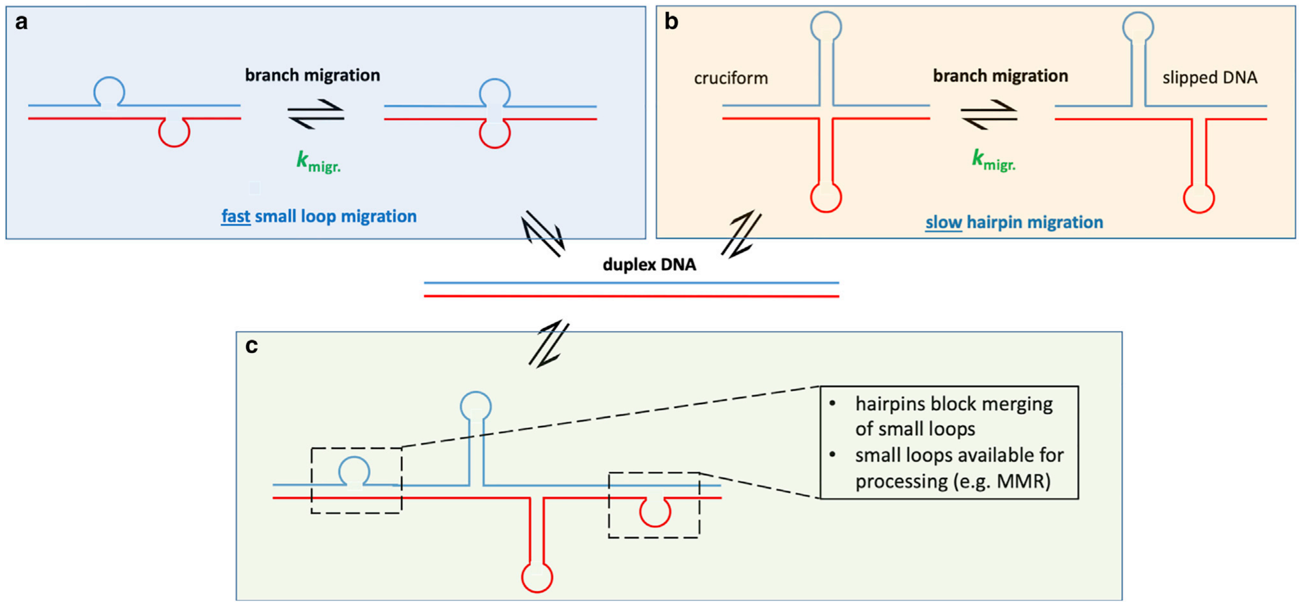

FIGURE 9 Branch migration of small unpaired loops and stable hairpins. (*a*) Small loops can move freely across a duplex composed of fully-paired repeats. (*b*) Large hairpins can migrate more slowly than small loops. (*c*) Large hairpins might act as roadblocks for small loops, preventing loops on opposite strands from merging and increasing the time available for processing of small loops (e.g., by mismatch repair).

K was chosen to be 10, HaMMy took up to 3 hours to provide the results on a laptop with operating system Windows 10 Pro and an Intel Core i5-6200 processor. This is attributed to the size of the stitched trajectories (up to 45,000 data points), which is close to the limit of datapoints that can be analyzed via HaMMy.(28) Overall, when the unstitched trajectories were analyzed, the computational time reduced significantly across the whole dataset, typically taking half of the time required for the stitched ones.

After running, the program outputs a file containing the idealized FRET trajectories with the intensity values of each transition and the idealized FRET state. The output files were imported in MASH-FRET 1.3.1 in the "Transition analysis" tab to build TDPs and obtain the optimal number of states K_{opt}.(29) In the TDPs, the transitions from one FRET state to another were counted and evenly spaced out in 100 bins between 0 and 1. The TDP was further convoluted to obtain a smooth plot ($\sigma^2 = 0.0005$).(28,70) To perform model selection, the TDPs were fitted to a mixture of $K \times (K - 1)$ isotropic 2D Gaussians. FRET values were derived from the Gaussian means and the associated errors (in parentheses) from the average Gaussian sample standard deviations. The value of K_{opt} was inferred via ML-BIC optimization of models comprising $K = 1$ to 10 states with 10 initializations. Overall, a total of 10 TDPs (five for each system) were fitted, and the results were compared to determine the most likely model.

Finally, the analyses were carried out using MATLAB R2020b on a laptop with operating system Windows 10 Pro and an Intel Core i5-6200 processor. Trace analysis was performed using HaMMy v4.0(28) and MASH-FRET 1.3.1.(29)

Dwell time cumulative probability distributions

CPDs were plotted using the cumulative dwell times histograms provided by MASH-FRET after TDP clustering.(29) The cumulative counts were imported to Origin v2020b and plotted as histograms. The rate coefficients were then recovered by fitting the cumulative counts to either single exponential decay (ExpDec1) or biexponential decay (ExpDec2) using nonlinear curve fitting (Levenberg-Marquardt). Average dwell times ($\tau_{av.}$) for biexponential fits were calculated by weighting the dwell times (τ) by the amplitudes (A): $\tau_{av.} = A_1\tau_1 + A_2\tau_2$.

Coarse-grained modeling

We made use of the well-tempered metadynamics method for enhanced sampling of rare events through the PLUMED library,(71,72) which is interfaced with the LAMMPS code through its PLUMED package. In particular, we used the LAMMPS version from July 2, 2021, and PLUMED version 2.7.1.

A bias in two independent collective variables measuring the approximate number of basepairs in specific locations was applied. One collective variable measured the number of basepairs that were to form in the hairpin loop and in the arm receiving the slipped-out sequence during a slippage event. The other collective variable consisted of the number of basepairs that would denature in the hairpin loop and in the other arm. Both collective variables were defined through a PLUMED contact map function, which measures the proximity of specific entities through smooth, but sharply decaying step functions. This entails that the number of basepairs is not integer valued and only approximate to the number of actual hydrogen bonds. Nevertheless, it gives a well-defined and clear control handle for the biasing algorithm and characterization of the individual states.

In the initial state the value of both collective variables is minimal and increases during the slippage event. The metadynamics algorithm biases the free energy landscape by adding gradually small Gaussian bias potentials, therefore forcing the system to move away from the energetically more favorable states and towards other, less favorable states that would normally not be accessible during realistic simulation times. Examples of these states include the intermediate states in Fig. 7 *k* and *l* between the initial and fully slipped-out final state. By recording the applied bias over time, running the metadynamics algorithm until convergence and tallying the recorded bias afterward, it is possible to extract an accurate picture of the free energy landscape of the unbiased system as the inverse sum of all

applied bias potentials. The fact that our free energy values are fully compatible with the experimentally observed transition rate of $O(1)s^{-1}$ provides strong evidence that the detected values are indeed realistic. All simulations were run at a temperature T $=$ 300 K using a metadynamics bias factor $\gamma = 8.0$ and different random seeds in a Langevin thermostat to sample independent trajectories. The electrostatic interaction is modelled by a Debye-Hückel approximation with implicit ions. Although the salt concentration of the presented runs is 0.2 M, the oxDNA2 model has been parameterized to minimize the difference between the observed melting temperatures T_m in oxDNA2 and the SantaLucia nearest-neighbor model for a range of salt concentrations between 0.1 and 0.5 M. This leads to a difference ΔT_m of around 0.25 K.(27)

The metadynamics algorithm was deemed as having converged when frequent transitions between the two basins of the initial and final state were observed and the height of the applied Gaussian bias potentials (as controlled through the algorithm) had significantly tapered off. For more information on the metadynamics method, we refer to a recent review.(73)

Data availability

The data that support the findings of this study are available from the University of Glasgow data repository Enlighten at http://dx.doi.org/10.5525/gla.researchdata.1337.

SUPPORTING MATERIAL

Supporting material can be found online at https://doi.org/10.1016/j.bpr.2022.100070.

AUTHOR CONTRIBUTIONS

S.W.M., O.H., and S.B. wrote the manuscript. T.H. performed the smFRET experiments. S.B. analyzed the smFRET data with assistance from T.H. and S.W.M. O.H. performed and analyzed the coarse-grained modeling.

ACKNOWLEDGMENTS

We are grateful to Mélodie Hadzic and Richard Börner for help with implementing MASH-FRET and to Darren Monckton for helpful discussions. T.H. was supported by the China Scholarship Council and by the Biotechnology and Biological Sciences Research Council (Grant No. BB/T003197/1). O.H. acknowledges support from the EPSRC Early Career Research Software Engineer Fellowship Scheme (Grant No. EP/N019180/2). For the purpose of complying with UKRI's open access policy, the authors have applied a Creative Commons Attribution (CC BY) license to any Author Accepted Manuscript version arising from this submission. This work used the ARCHIE-WeSt High-Performance Computer (www.archie-west.ac.uk) based at the University of Strathclyde.

DECLARATION OF INTERESTS

The authors declare no competing interests.

REFERENCES

1. Khristich, A. N., and S. M. Mirkin. 2020. On the wrong DNA track: Molecular mechanisms of repeat-mediated genome instability. *J. Biol. Chem.* 295:4134–4170. https://doi.org/10.1074/jbc.REV119.007678.
2. Liu, G., X. Chen, ..., M. Leffak. 2010. Replication-dependent instability at (CTG)•(CAG) repeat hairpins in human cells. *Nat. Chem. Biol.* 6:652–659. https://doi.org/10.1038/nchembio.416.
3. Kim, J. C., S. T. Harris, ..., S. M. Mirkin. 2017. The role of break-induced replication in large-scale expansions of $(CAG)_n/(CTG)_n$ repeats. *Nat. Struct. Mol. Biol.* 24:55–60. https://doi.org/10.1038/nsmb.3334.
4. Gomes-Pereira, M., M. T. Fortune, ..., D. G. Monckton. 2004. Pms2 is a genetic enhancer of trinucleotide CAG•CTG repeat somatic mosaicism: implications for the mechanism of triplet repeat expansion. *Hum. Mol. Genet.* 13:1815–1825. https://doi.org/10.1093/hmg/ddh186.
5. Iyer, R. R., and A. Pluciennik. 2021. DNA mismatch repair and its role in Huntington's disease. *J. Huntington's Dis.* 10:75–94. https://doi.org/10.3233/jhd-200438.
6. Zhao, X. N., and K. Usdin. 2015. The repeat expansion diseases: the dark side of DNA repair. *DNA Repair.* 32:96–105. https://doi.org/10.1016/j.dnarep.2015.04.019.
7. Schmidt, M. H. M., and C. E. Pearson. 2016. Disease-associated repeat instability and mismatch repair. *DNA Repair.* 38:117–126. https://doi.org/10.1016/j.dnarep.2015.11.008.
8. Iyer, R. R., A. Pluciennik, ..., R. D. Wells. 2015. DNA triplet repeat expansion and mismatch repair. *Annu. Rev. Biochem.* 84:199–226. https://doi.org/10.1146/annurev-biochem-060614-034010.
9. Trost, B., W. Engchuan, ..., R. K. C. Yuen. 2020. Genome-wide detection of tandem DNA repeats that are expanded in autism. *Nature.* 586:80–86. https://doi.org/10.1038/s41586-020-2579-z.
10. Mitra, I., B. Huang, ..., M. Gymrek. 2021. Patterns of de novo tandem repeat mutations and their role in autism. *Nature.* 589:246–250. https://doi.org/10.1038/s41586-020-03078-7.
11. McMurray, C. T. 1999. DNA secondary structure: A common and causative factor for expansion in human disease. *Proc. Natl. Acad. Sci. USA.* 96:1823–1825. https://doi.org/10.1073/pnas.96.5.1823.
12. Gacy, A. M., G. Goellner, C. T. McMurray..., 1995. Trinucleotide repeats that expand in human disease form hairpin structures in vitro. *Cell.* 81:533–540. https://doi.org/10.1016/0092-8674(95)90074-8.
13. Pearson, C. E., and R. R. Sinden. 1996. Alternative structures in duplex DNA formed within the trinucleotide repeats of the myotonic dystrophy and fragile X loci. *Biochemistry.* 35:5041–5053. https://doi.org/10.1021/bi9601013.
14. Gacy, A. M., and C. T. McMurray. 1998. Influence of hairpins on template reannealing at trinucleotide repeat duplexes: A model for slipped DNA. *Biochemistry.* 37:9426–9434. https://doi.org/10.1021/bi980157s.
15. Pearson, C. E., M. Tam, K. Nichol..., 2002. Slipped-strand DNAs formed by long (CAG)•(CTG) repeats: slipped-out repeats and slip-out junctions. *Nucleic Acids Res.* 30:4534–4547. https://doi.org/10.1093/nar/gkf572.
16. Polyzos, A. A., and C. T. McMurray. 2017. Close encounters: Moving along bumps, breaks, and bubbles on expanded trinucleotide tracts. *DNA Repair.* 56:144–155. https://doi.org/10.1016/j.dnarep.2017.06.017.
17. López Castel, A., J. D. Cleary, and C. E. Pearson. 2010. Repeat instability as the basis for human diseases and as a potential target for therapy. *Nat. Rev. Mol. Cell Biol.* 11:165–170. https://doi.org/10.1038/nrm2854.
18. Usdin, K. 2008. The biological effects of simple tandem repeats: Lessons from the repeat expansion diseases. *Genome Res.* 18:1011–1019. https://doi.org/10.1101/gr.070409.107.
19. Hu, T., M. J. Morten, and S. W. Magennis. 2021. Conformational and migrational dynamics of slipped-strand DNA three-way junctions containing trinucleotide repeats. *Nat. Commun.* 12:204. https://doi.org/10.1038/s41467-020-20426-3.

20. Völker, J., N. Makube, G. E. Plum, H. H. Klump, and K. J. Breslauer. 2002. Conformational energetics of stable and metastable states formed by DNA triplet repeat oligonucleotides: Implications for triplet expansion diseases. *Proc. Natl. Acad. Sci. USA.* 99:14700–14705. https://doi.org/10.1073/pnas.222519799.

21. Völker, J., V. Gindikin, H. H. Klump, G. E. Plum, and K. J. Breslauer. 2012. Energy landscapes of dynamic ensembles of rolling triplet repeat bulge loops: implications for DNA expansion associated with disease states. *J. Am. Chem. Soc.* 134:6033–6044. https://doi.org/10.1021/ja3010896.

22. Völker, J., and K. J. Breslauer. 2021. Differential repair enzyme-substrate selection within dynamic DNA energy landscapes. *Q. Rev. Biophys.* 55:e1. https://doi.org/10.1017/S0033583521000093.

23. Mitchell, M. L., M. P. Leveille, B. Cannon…, 2018. Sequence-dependent effects of monovalent cations on the structural dynamics of trinucleotide-repeat DNA hairpins. *J. Phys. Chem. B.* 122:11841–11851. https://doi.org/10.1021/acs.jpcb.8b07994.

24. Ni, C.-W., Y.-J. Wei, I.-R. Lee…, 2019. Long-range hairpin slippage reconfiguration dynamics in trinucleotide repeat sequences. *J. Phys. Chem. Lett.* 10:3985–3990. https://doi.org/10.1021/acs.jpclett.9b01524.

25. Xu, P., F. Pan, K. Weninger…, 2020. Dynamics of strand slippage in DNA hairpins formed by CAG repeats: roles of sequence parity and trinucleotide interrupts. *Nucleic Acids Res.* 48:2232–2245. https://doi.org/10.1093/nar/gkaa036.

26. Ouldridge, T. E., A. A. Louis, and J. P. K. Doye. 2011. Structural, mechanical, and thermodynamic properties of a coarse-grained DNA model. *J. Chem. Phys.* 134:085101. https://doi.org/10.1063/1.3552946.

27. Snodin, B. E. K., F. Randisi, …, J. P. K. Doye. 2015. Introducing improved structural properties and salt dependence into a coarse-grained model of DNA. *J. Chem. Phys.* 142:234901. https://doi.org/10.1063/1.4921957.

28. McKinney, S. A., C. Joo, and T. Ha. 2006. Analysis of single-molecule FRET trajectories using hidden Markov modeling. *Biophys. J.* 91:1941–1951. https://doi.org/10.1529/biophysj.106.082487.

29. Hadzic, M. C. A. S., R. Börner, …, R. K. O. Sigel. 2018. Reliable state identification and state transition detection in fluorescence intensity-based single-molecule Förster resonance energy-transfer data. *J. Phys. Chem. B.* 122:6134–6147. https://doi.org/10.1021/acs.jpcb.7b12483.

30. Blanco, M., and N. G. Walter. 2010. Analysis of complex single-molecule FRET time trajectories. *In* Methods in Enzymology, Methods in Enzymology, Vol 472: Single Molecule Tools, Pt A: Fluorescence Based Approaches N. G. Walter, ed. Elsevier, pp. 153–178. https://doi.org/10.1016/s0076-6879(10)72011-5.

31. Baltierra-Jasso, L. E., M. J. Morten, …, S. W. Magennis. 2015. Crowding-induced hybridization of single DNA hairpins. *J. Am. Chem. Soc.* 137:16020–16023. https://doi.org/10.1021/jacs.5b11829.

32. Sabir, T., A. Toulmin, S. W. Magennis…, 2012. Branchpoint expansion in a fully complementary three-way DNA junction. *J. Am. Chem. Soc.* 134:6280–6285. https://doi.org/10.1021/ja211802z.

33. Toulmin, A., L. E. Baltierra-Jasso, S. W. Magennis…, 2017. Conformational heterogeneity in a fully complementary DNA three-way junction with a GC-rich branchpoint. *Biochemistry.* 56:4985–4991. https://doi.org/10.1021/acs.biochem.7b00677.

34. Plimpton, S. 1995. Fast parallel algorithms for short-range molecular-dynamics. *J. Comput. Phys.* 117:1–19. https://doi.org/10.1006/jcph.1995.1039.

35. Henrich, O., Y. A. Gutiérrez Fosado, …, T. E. Ouldridge. 2018. Coarse-grained simulation of DNA using LAMMPS An implementation of the oxDNA model and its applications. *Eur. Phys. J. E Soft Matter.* 41:57. https://doi.org/10.1140/epje/i2018-11669-8.

36. Tsukanov, R., T. E. Tomov, …, E. Nir. 2013. Detailed study of DNA hairpin dynamics using single-molecule fluorescence assisted by DNA origami. *J. Phys. Chem. B.* 117:11932–11942. https://doi.org/10.1021/jp4059214.

37. Woodside, M. T., W. M. Behnke-Parks, …, S. M. Block. 2006. Nanomechanical measurements of the sequence-dependent folding landscapes of single nucleic acid hairpins. *Proc. Natl. Acad. Sci. USA.* 103:6190–6195. https://doi.org/10.1073/pnas.0511048103.

38. Liu, Y., J. Park, K. A. Dahmen, Y. R. Chemla, and T. Ha. 2010. A comparative study of multivariate and univariate hidden Markov modelings in time-binned single-molecule FRET data analysis. *J. Phys. Chem. B.* 114:5386–5403. https://doi.org/10.1021/jp9057669.

39. Rabiner, L. R. 1989. A tutorial on hidden Markov-models and selected applications in speech recognition. *Proc. IEEE.* 77:257–286. https://doi.org/10.1109/5.18626.

40. Sgouralis, I., S. Madaan, …, S. Pressé. 2019. A Bayesian nonparametric approach to single molecule Förster resonance energy transfer. *J. Phys. Chem. B.* 123:675–688. https://doi.org/10.1021/acs.jpcb.8b09752.

41. Kilic, Z., I. Sgouralis, …, S. Pressé. 2021. Extraction of rapid kinetics from smFRET measurements using integrative detectors. *Cell Rep. Phys. Sci.* 2:100409. https://doi.org/10.1016/j.xcrp.2021.100409.

42. Lerner, E., A. Barth, …, S. Weiss. 2021. FRET-based dynamic structural biology: Challenges, perspectives and an appeal for open-science practices. *Elife.* 10:e60416. https://doi.org/10.7554/eLife.60416.

43. Pirchi, M., R. Tsukanov, …, E. Nir. 2016. Photon-by-photon hidden Markov model analysis for microsecond single-molecule FRET kinetics. *J. Phys. Chem. B.* 120:13065–13075. https://doi.org/10.1021/acs.jpcb.6b10726.

44. Schmid, S., M. Götz, and T. Hugel. 2016. Single-molecule analysis beyond dwell times: demonstration and assessment in and out of equilibrium. *Biophys. J.* 111:1375–1384. https://doi.org/10.1016/j.bpj.2016.08.023.

45. Schmid, S., and T. Hugel. 2018. Efficient use of single molecule time traces to resolve kinetic rates, models and uncertainties. *J. Chem. Phys.* 148:123312. https://doi.org/10.1063/1.5006604.

46. Hon, J., and R. L. Gonzalez, Jr. 2019. Bayesian-estimated hierarchical HMMs enable robust analysis of single-molecule kinetic heterogeneity. *Biophys. J.* 116:1790–1802. https://doi.org/10.1016/j.bpj.2019.02.031.

47. Götz, M., A. Barth, …, S. Schmid. 2021. Inferring kinetic rate constants from single-molecule FRET trajectories – a blind benchmark of kinetic analysis tools. Preprint at bioRxiv. https://doi.org/10.1101/2021.11.23.469671.

48. Thomsen, J., M. B. Sletfjerding, S. B. Jensen, S. Stella, B. Paul, M. G. Malle, G. Montoya, T. C. Petersen, and N. S. Hatzakis. 2020. DeepFRET, a software for rapid and automated single-molecule FRET data classification using deep learning. *Elife.* 9:e60404. https://doi.org/10.7554/eLife.60404.

49. Wasserman, L. 2000. Bayesian model selection and model averaging. *J. Math. Psychol.* 44:92–107. https://doi.org/10.1006/jmps.1999.1278.

50. Bronson, J. E., J. M. Hofman, …, C. H. Wiggins. 2010. Graphical models for inferring single molecule dynamics. *BMC Bioinf.* 11:S2. https://doi.org/10.1186/1471-2105-11-s8-s2.

51. Barducci, A., G. Bussi, and M. Parrinello. 2008. Well-tempered metadynamics: A smoothly converging and tunable free-energy method. *Phys. Rev. Lett.* 100:020603. https://doi.org/10.1103/PhysRevLett.100.020603.

52. Hyeon, C., J. Lee, J. Yoon, S. Hohng, and D. Thirumalai. 2012. Hidden complexity in the isomerization dynamics of Holliday junctions. *Nat. Chem.* 4:907–914. https://doi.org/10.1038/nchem.1463.

53. Slean, M. M., K. Reddy, …, C. E. Pearson. 2013. Interconverting conformations of slipped-DNA junctions formed by trinucleotide

repeats affect repair outcome. *Biochemistry.* 52:773–785. https://doi.org/10.1021/bi301369.

54. Amrane, S., B. Saccà, M. Mills, J. L. Mergny…, 2005. Length-dependent energetics of $(CTG)_n$ and $(CAG)_n$ trinucleotide repeats. *Nucleic Acids Res.* 33:4065–4077. https://doi.org/10.1093/nar/gki716.

55. Petruska, J., M. J. Hartenstine, and M. F. Goodman. 1998. Analysis of strand slippage in DNA polymerase expansions of CAG/CTG triplet repeats associated with neurodegenerative disease. *J. Biol. Chem.* 273:5204–5210. https://doi.org/10.1074/jbc.273.9.5204.

56. Hartenstine, M. J., M. F. Goodman, and J. Petruska. 2000. Base stacking and even/odd behavior of hairpin loops in DNA triplet repeat slippage and expansion with DNA polymerase. *J. Biol. Chem.* 275:18382–18390. https://doi.org/10.1074/jbc.275.24.18382.

57. Panyutin, I. G., and P. Hsieh. 1993. Formation of a single base mismatch impedes spontaneous DNA branch migration. *J. Mol. Biol.* 230:413–424. https://doi.org/10.1006/jmbi.1993.1159.

58. Baltierra-Jasso, L. E., M. J. Morten, and S. W. Magennis. 2018. Sub-ensemble monitoring of DNA strand displacement using multiparameter single-molecule FRET. *ChemPhysChem.* 19:551–555. https://doi.org/10.1002/cphc.201800012.

59. Vonsattel, J. P. G. …, Genetic Modifiers of Huntington's Disease GeM-HD Consortium. 2019. CAG repeat not polyglutamine length determines timing of Huntington's disease onset. *Cell.* 178:887–900.e14. https://doi.org/10.1016/j.cell.2019.06.036.

60. Monckton, D. G. 2021. The contribution of somatic expansion of the CAG repeat to symptomatic development in Huntington's disease: a historical perspective. *J. Huntington's Dis.* 10:7–33. https://doi.org/10.3233/jhd-200429.

61. Pluciennik, A., V. Burdett, …, P. Modrich. 2013. Extrahelical (CAG)/(CTG) triplet repeat elements support proliferating cell nuclear antigen loading and MutL alpha endonuclease activation. *Proc. Natl. Acad. Sci. USA.* 110:12277–12282. https://doi.org/10.1073/pnas.1311325110.

62. Kadyrova, L. Y., V. Gujar, …, F. A. Kadyrov. 2020. Human MutL gamma, the MLH1-MLH3 heterodimer, is an endonuclease that promotes DNA expansion. *Proc. Natl. Acad. Sci. USA.* 117:3535–3542. https://doi.org/10.1073/pnas.1914718117.

63. Tomé, S., I. Holt, …, G. Gourdon. 2009. MSH2 ATPase domain mutation affects CTG•CAG repeat instability in transgenic mice. *PLoS Genet.* 5:e1000482. https://doi.org/10.1371/journal.pgen.1000482.

64. Panigrahi, G. B., R. Lau, …, C. E. Pearson. 2005. Slipped (CTG)•(CAG) repeats can be correctly repaired, escape repair or undergo error-prone repair. *Nat. Struct. Mol. Biol.* 12:654–662. https://doi.org/10.1038/nsmb959.

65. Axford, M. M., Y.-H. Wang, C. E. Pearson…, 2013. Detection of slipped-DNAs at the trinucleotide repeats of the myotonic dystrophy type I disease locus in patient tissues. *PLoS Genet.* 9:e1003866. https://doi.org/10.1371/journal.pgen.1003866.

66. Kaplan, S., S. Itzkovitz, and E. Shapiro. 2007. A Universal Mechanism Ties Genotype to Phenotype in Trinucleotide Diseases. *PLoS Comput. Biol.* 3:e235. https://doi.org/10.1371/journal.pcbi.0030235.

67. Kacher, R., F.-X. Lejeune, …, A. Durr. 2021. Propensity for somatic expansion increases over the course of life in Huntington disease. *Elife.* 10:e64674. https://doi.org/10.7554/eLife.64674.

68. Lang, W. H., J. E. Coats, C. T. McMurray…, 2011. Conformational trapping of Mismatch Recognition Complex MSH2/MSH3 on repair-resistant DNA loops. *Proc. Natl. Acad. Sci. USA.* 108:E837–E844. https://doi.org/10.1073/pnas.1105461108.

69. Holden, S. J., S. Uphoff, …, A. N. Kapanidis. 2010. Defining the limits of single-molecule FRET resolution in TIRF microscopy. *Biophys. J.* 99:3102–3111. https://doi.org/10.1016/j.bpj.2010.09.005.

70. König, S. L. B., M. Hadzic, …, R. K. O. Sigel. 2013. BOBA FRET: bootstrap-based analysis of single-molecule FRET data. *PLoS One.* 8:e84157. https://doi.org/10.1371/journal.pone.0084157.

71. PLUMED consortium, Bussi, G., …, D. Branduardi 2019. Promoting transparency and reproducibility in enhanced molecular simulations. *Nat. Methods.* 16:670–673. https://doi.org/10.1038/s41592-019-0506-8.

72. Tribello, G. A., M. Bonomi, …, G. Bussi. 2014. PLUMED 2: New feathers for an old bird. *Comput. Phys. Commun.* 185:604–613. https://doi.org/10.1016/j.cpc.2013.09.018.

73. Bussi, G., and A. Laio. 2020. Using metadynamics to explore complex free-energy landscapes. *Nat. Rev. Phys.* 2:200–212. https://doi.org/10.1038/s42254-020-0153-0.

Biophysical Reports, Volume *2*

Supplemental information

Heterogeneous migration routes of DNA triplet repeat slip-outs

Simona Bianco, Tianyu Hu, Oliver Henrich, and Steven W. Magennis

Supporting Material

Oligonucleotide sequences

Supplemental Tables 1-8

Supplemental Figures 1-18

Oligonucleotide sequences

a) **DNA hairpin**

Donor strand: 5' /Biotin/ TGG CGA CGG CAG CGA GGC TTA GCG GCA AAA AAA AAA AAA AAA AAA AAA AAA AAA AAA AGC CGC **X**

where X = T-Alexa488

Acceptor strand: 5' GCC TCG C**Y**G CCG TCG CCA

where Y = T-Cy5

b) **Static 3WJs with $(CAG)_{10}$ slipouts**

X = C-Cy5 and Y = T-Alexa488

S-CAG-1

Top: 5’ AGT GGT CAG ACG CAG CAG CAG CAG CAG CAG CAG CAG CAG **X**AG CTC GGC ACT CGT GAT TTG GTC

Bottom: 5' /Biotin/ GAC CAA ATC ACG AGT GCC GAG CGT C**Y**G ACC ACT

S-CAG-2

Top: 5' AGT GGT CAG ACG ATG CAG CAG CAG CAG CAG CAG CAG CAG **X**AG CAG CTC ACT CGT GAT TTG GTC

Bottom: 5' / Biotin/ GAC CAA ATC ACG AGT GAG CAT CGT C**Y**G ACC ACT

S-CAG-3

Top: 5' AGT GGT CAG ACG ACT ATG CAG CAG CAG CAG CAG CAG CAG **X**AG CAG CAG CTC CGT GAT TTG GTC

Bottom: 5' /Biotin/ GAC CAA ATC ACG GAG CAT AGT CGT C**Y**G ACC ACT

S-CAG-4

Top: 5' AGT GGT CAG ACG ACT GGC ATG CAG CAG CAG CAG CAG CAG **X**AG CAG CAG CAG CGT GAT TTG GTC

Bottom: 5' /Biotin/ GAC CAA ATC ACG CAT GCC AGT CGT C**Y**G ACC ACT

Fully complementary static 3WJ

Top: 5' AGT GGT CAG ACG ACT ATG CTG CTG CTG CTG CTG CAG CAG **X**AG CAG CAG CTC CGT GAT TTG GTC

Bottom: 5' /Biotin/ GAC CAA ATC ACG GAG CAT AGT CGT C**Y**G ACC ACT

c) **Mobile 3WJs**

X = C-Cy5 and Y = T-Alexa488

$(CAG)_{10}$

Top: 5' AGT GGT CAG ACG CAG CAG CAG CAG CAG CAG CAG CAG CAG **X**AG CAG CAG CAG CGT GAT TTG GTC

$(CAG)_{40}$

Top: 5' AGT GGT CAG ACG CAG CAG CAG CAG CAG CAG CAG CAG CAG CAG CAG CAG CAG CAG CAG CAG CAG CAG CAG CAG CAG CAG CAG CAG CAG CAG CAG CAG CAG CAG CAG CAG CAG CAG CAG CAG CAG CAG CAG **X**AG CAG CAG CAG CGT GAT TTG GTC

Bottom: 5’ /Biotin/ GAC CAA ATC ACG CTG CTG CTG CGT C**Y**G ACC ACT

$(CTG)_{30}$

Top: 5' AGT GGT CAG ACG CTG CTG CTG CTG CTG CTG CTG CTG CTG CTG CTG CTG CTG CTG CTG CTG CTG CTG CTG CTG CTG CTG CTG CTG CTG CTG CTG CTG CTG **X**TG CTG CTG CTG CGT GAT TTG GTC

Bottom: 5' /Biotin/ GAC CAA ATC ACG CAG CAG CAG CGT C**Y**G ACC ACT

Supplemental Tables

Table S1: FRET states obtained from HaMMy after TDP fitting and ML-BIC optimisation of the mobile $(CAG)_{10}$ stitched trajectory.

FRET State	1	2	3	4	5	6	7	8	9
6	0.07	0.29	0.46	0.61	0.82	0.92			
7	0.07	0.27	0.44	0.61	0.82	0.92			
8	0.07	0.27	0.44	0.60	0.72	0.84	0.93		
9	0.06	0.16	0.31	0.45	0.59	0.73	0.84	0.93	
10	0.05	0.07	0.16	0.29	0.46	0.60	0.74	0.86	0.93

Table S2: FRET states obtained from HaMMy after TDP fitting and ML-BIC optimisation of the mobile $(CTG)_{30}$ stitched trajectory.

FRET State	1	2	3	4	5	6	7	8	9
6	0.10	0.26	0.40	0.58	0.76	0.90			
7	0.09	0.22	0.38	0.45	0.61	0.78	0.91		
8	0.09	0.21	0.32	0.39	0.55	0.70	0.83	0.91	
9	0.09	0.18	0.30	0.40	0.49	0.61	0.73	0.85	0.92
10	0.08	0.15	0.27	0.41	0.46	0.60	0.74	0.85	0.92

Table S3: BIC values determined across the range of states 2-10 by TDP clustering after HaMMy analysis of the unstitched trajectories of mobile $(CAG)_{10}$, $(CTG)_{30}$ and $(CAG)_{40}$. The most sufficient state configuration (K_{opt}) is in italics.

**$(CAG)_{10}$**									
	BIC(2)	BIC(3)	BIC(4)	BIC(5)	BIC(6)	BIC(7)	BIC(8)	BIC(9)	BIC(10)
$K = 6$	1.702	1.1429	0.64105	0.58144	*0.40066*	0.53732	0.43453	0.48167	0.60959
$K = 7$	1.6338	1.0504	0.80262	0.70353	*0.57969*	0.6092	0.64236	0.68827	0.78373
$K = 8$	1.684	1.0753	0.53509	0.59855	*0.32549*	0.33816	0.33816	0.40795	0.52494
$K = 9$	1.6511	1.0877	0.59207	0.5639	*0.40379*	0.41682	0.43915	0.51133	0.6382
$K = 10$	1.7314	1.1724	0.67285	0.60734	0.51146	***0.43501***	0.48877	0.54963	0.65184
**$(CTG)_{30}$**									
	BIC(2)	BIC(3)	BIC(4)	BIC(5)	BIC(6)	BIC(7)	BIC(8)	BIC(9)	BIC(10)
$K = 6$	1.6982	1.1094	0.54029	0.26208	*0.22135*	0.34056	0.53085	0.56554	0.79665
$K = 7$	1.7097	1.2589	0.73208	0.5597	*0.4988*	0.52362	0.70553	0.78155	1.0309
$K = 8$	1.7231	1.1149	0.52509	0.47601	*0.30891*	0.30936	0.56456	0.61878	0.80666
$K = 9$	1.6307	1.204	0.79798	0.59009	*0.5285*	0.54392	0.80136	0.78663	1.0129
$K = 10$	1.6445	1.2276	0.78701	*0.5693*	0.58422	0.68499	0.80706	0.93324	1.0816
**$(CAG)_{40}$**									
	BIC(2)	BIC(3)	BIC(4)	BIC(5)	BIC(6)	BIC(7)	BIC(8)	BIC(9)	BIC(10)
$K = 6$	1.4934	1.2911	0.94305	0.8062	*0.72884*	0.82799	0.81475	0.85936	0.94443
$K = 7$	1.455	1.2176	0.934	0.80595	0.78686	*0.78487*	0.82058	0.87962	0.8991
$K = 8$	1.5122	1.2672	0.94404	0.81272	*0.74124*	0.79108	0.77801	0.88824	0.93991
$K = 9$	1.4663	1.229	0.9528	0.87264	*0.75532*	0.79861	0.78273	0.91051	0.95775
$K = 10$	1.5381	1.2821	0.95305	0.8125	*0.73743*	0.79946	0.806	0.90872	0.93585

Table S4: FRET states obtained from HaMMy after TDP fitting and ML-BIC optimisation of the mobile $(CAG)_{10}$ unstitched trajectories.

FRET State	1	2	3	4	5	6	7
6	0.08	0.26	0.43	0.62	0.79	0.93	
7	0.08	0.28	0.44	0.62	0.80	0.93	
8	0.08	0.26	0.42	0.63	0.80	0.93	
9	0.07	0.25	0.41	0.62	0.80	0.93	
10	0.07	0.22	0.37	0.52	0.67	0.83	0.94

Table S5: FRET states obtained from HaMMy after TDP fitting and ML-BIC optimisation of the mobile $(CTG)_{30}$ unstitched trajectories.

FRET State	1	2	3	4	5	6
6	0.09	0.21	0.37	0.59	0.78	0.92
7	0.09	0.22	0.41	0.59	0.78	0.92
8	0.09	0.22	0.41	0.60	0.77	0.92
9	0.09	0.24	0.43	0.59	0.79	0.92
10	0.09	0.30	0.52	0.75	0.91	

Table S6: FRET states obtained from HaMMy after TDP fitting and ML-BIC optimisation of the mobile $(CAG)_{40}$ unstitched trajectories.

FRET State	1	2	3	4	5	6	7
6	0.08	0.26	0.42	0.58	0.77	0.91	
7	0.07	0.20	0.37	0.50	0.64	0.80	0.92
8	0.08	0.24	0.42	0.57	0.76	0.91	
9	0.08	0.24	0.42	0.58	0.77	0.91	
10	0.08	0.23	0.42	0.58	0.77	0.91	

Table S7: Rate constants (s^{-1}) obtained from exponential fitting of the dwell time histograms obtained from the optimised TDP clusters of the unstitched trajectories of static $(CAG)_{10}$ 3WJs (S-CAG-1, S-CAG-2, S-CAG-3 and S-CAG-4), the fully complementary 3WJ and the DNA hairpin. The amplitudes corresponding to the rate constants of the biexponential fittings are provided in the brackets.

S-CAG-1<br>Monoexponential			
FRET states	S1	S2	S3
k_1	4.08	4.17	2.04
Biexponential			
k_1 (A_1 %)<br>k_2 (A_2 %)	12.49 (64.26%)<br>1.31 (35.74%)	5.74 (82.62%)<br>0.67 (17.38%)	2.9 (70.81%)<br>0.85 (29.19%)
S-CAG-2<br>Monoexponential			
FRET states	S1	S2	
k_1	3.72	2.71	
Biexponential			
k_1 (A_1 %)<br>k_2 (A_2 %)	8.5 (72.79%)<br>0.9 (27.21%)	4.5 (69.49%)<br>1.16 (30.51%)	
S-CAG-3<br>Monoexponential			
FRET states	S1	S2	S3
k_1	0.71	10.31	18.99
Biexponential			
k_1 (A_1 %)<br>k_2 (A_2 %)	3.8 (53.84%)<br>0.25 (46.16%)	27.9 (63.40%)<br>3.48 (36.60%)	21.34 (89.32%)<br>5.85 (10.68%)
S-CAG-4<br>Monoexponential			
FRET states	S1	S2	
k_1	0.26	12.68	
Biexponential			
k_1 (A_1 %)<br>k_2 (A_2 %)	1.82 (82.13%)<br>0.11 (17.87%)	20.64 (39.00%)<br>1.19 (61.00%)	
Fully complementary 3WJ<br>Monoexponential			
FRET states	S1	S2	
k_1	5.73	2.56	
Biexponential			
k_1 (A_1 %)<br>k_2 (A_2 %)	7.16 (79.10%)<br>0.26 (20.90%)	N/A<br>N/A	
Hairpin<br>Monoexponential			
FRET states	S1	S2	
k_1	2.46	2.16	
Biexponential			
k_1 (A_1 %)<br>k_2 (A_2 %)	4.18 (45.84%)<br>1.74 (54.16%)	2.33 (92.62%)<br>0.87(7.38%)	

Table S8: Rate constants (s^{-1}) obtained from exponential fitting of the dwell time histograms obtained from the optimised TDP clusters of the unstitched trajectories of mobile $(CAG)_{10}$, $(CTG)_{30}$ and $(CAG)_{40}$. The amplitudes corresponding to the rate constants of the biexponential fittings are provided in the brackets.

$(CAG)_{10}$ Monoexponential						
FRET states	S1	S2	S3	S4	S5	S6
k_1	2.24	1.99	6.59	5.87	1.36	1.37
Biexponential						
k_1 (A_1 %)	5.8 (53.71%)	7.98 (46.72%)	18.9 (65.29%)	33.32 (53.15%)	12.08 (45.11%)	4.04 (37.04%)
k_2 (A_2 %)	1.04 (46.29%)	1.07 (56.28%)	1.59(34.71%)	1.97 (46.85%)	0.85 (54.89%)	0.92 (62.96%)
$(CAG)_{30}$ Monoexponential						
FRET states	S1	S2	S3	S4	S5	S6
k_1	2.11	1.08	1.56	7.52	0.6	0.91
Biexponential						
k_1 (A_1 %)	12.2 (52.06%)	16.46 (27.31%)	5.93 (54.36%)	31.98 (65.52%)	4.57 (27.55%)	6.7 (27.74%)
k_2 (A_2 %)	0.99 (47.94%)	0.87 (72.69%)	0.59 (45.64%)	1.28 (34.48%)	0.44 (72.45%)	0.7 (72.26%)
$(CAG)_{40}$ Monoexponential						
FRET states	S1	S2	S3	S4	S5	S6
k_1	1.32	1.62	1.48	1.3	0.91	0.76
Biexponential						
k_1 (A_1 %)	10.48 (50.42%)	8.49 (39.29%)	12.94 (35.04%)	18.99 (19.64%)	2.84 (39.52%)	9.38 (23.62%)
k_2 (A_2 %)	0.72 (49.58%)	1.03 (60.71%)	1.06 (64.96%)	1.14 (80.36%)	0.57 (60.48%)	0.65 (79.38%)

Supplemental Figures

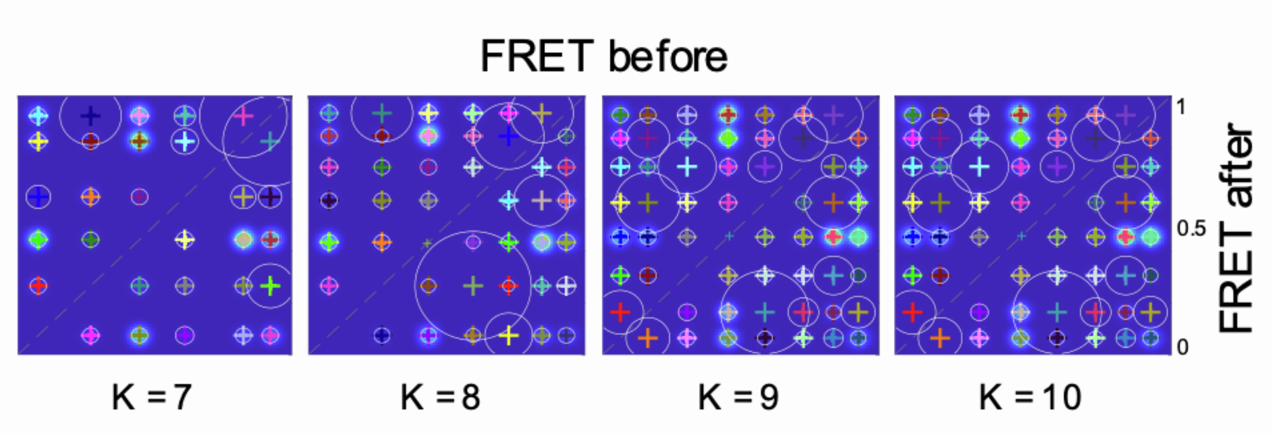

Figure S1. TDPs built from the stitched trajectories of mobile $(CAG)_{10}$ for $K = 7, 8, 9, 10$.

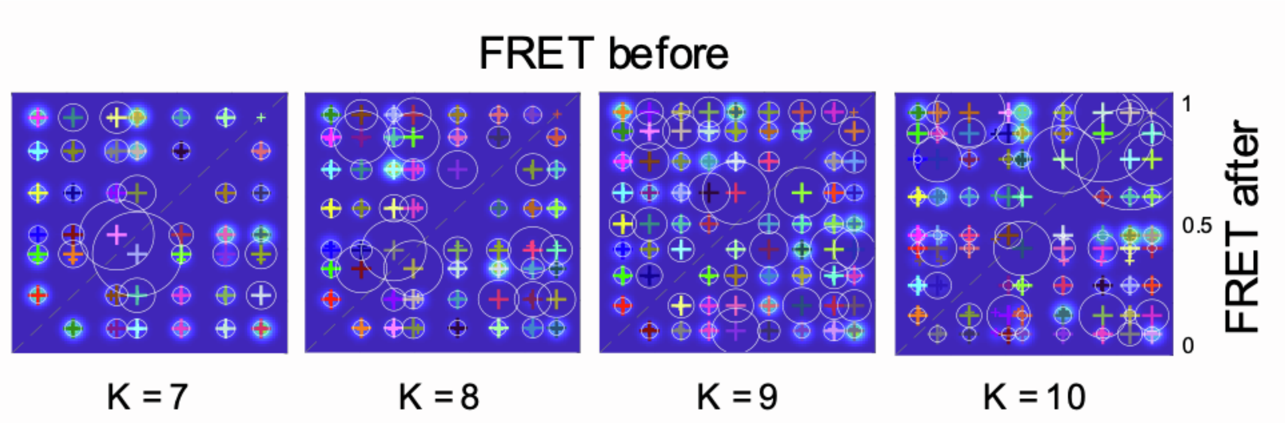

Figure S2. TDPs built from the stitched trajectories of mobile $(CTG)_{30}$ for $K = 7, 8, 9, 10$.

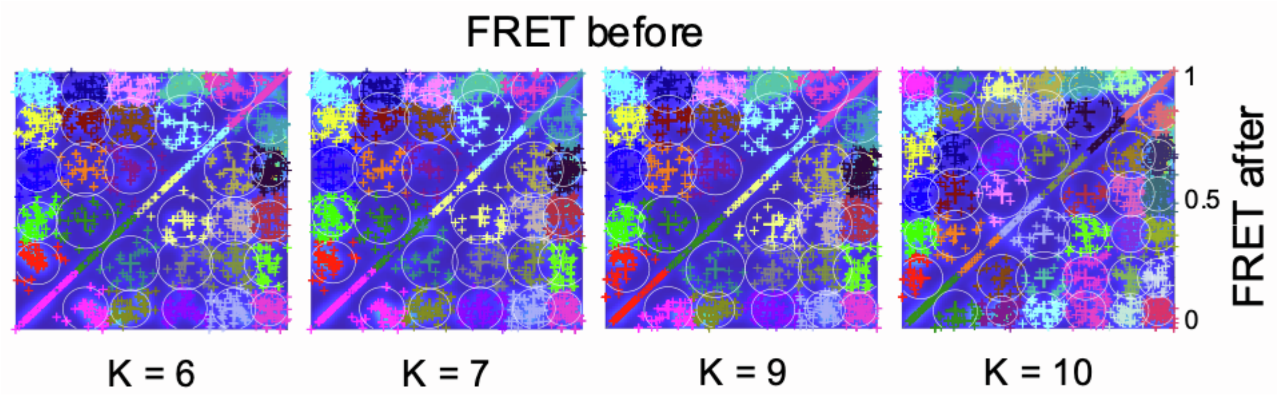

Figure S3. TDPs built from the unstitched trajectories of mobile $(CAG)_{10}$ for K = 6, 7, 9, 10.

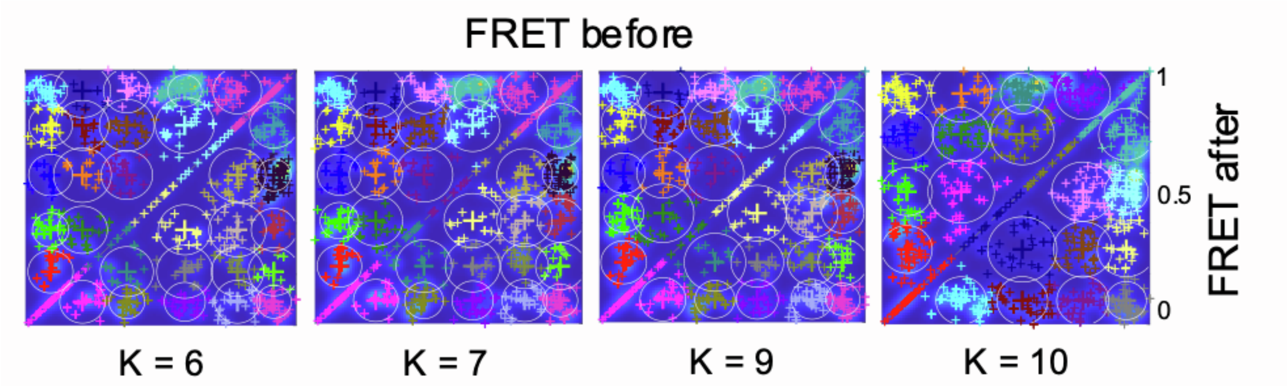

Figure S4. TDPs built from the unstitched trajectories of mobile $(CTG)_{30}$ for K = 6, 7, 9, 10.

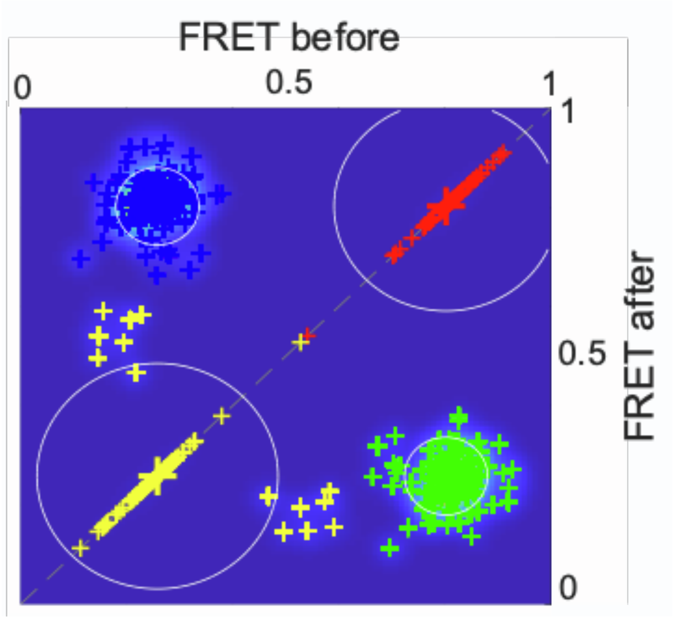

Figure S5. TDP from the unstitched trajectories of the DNA hairpin showing two-state dynamics.

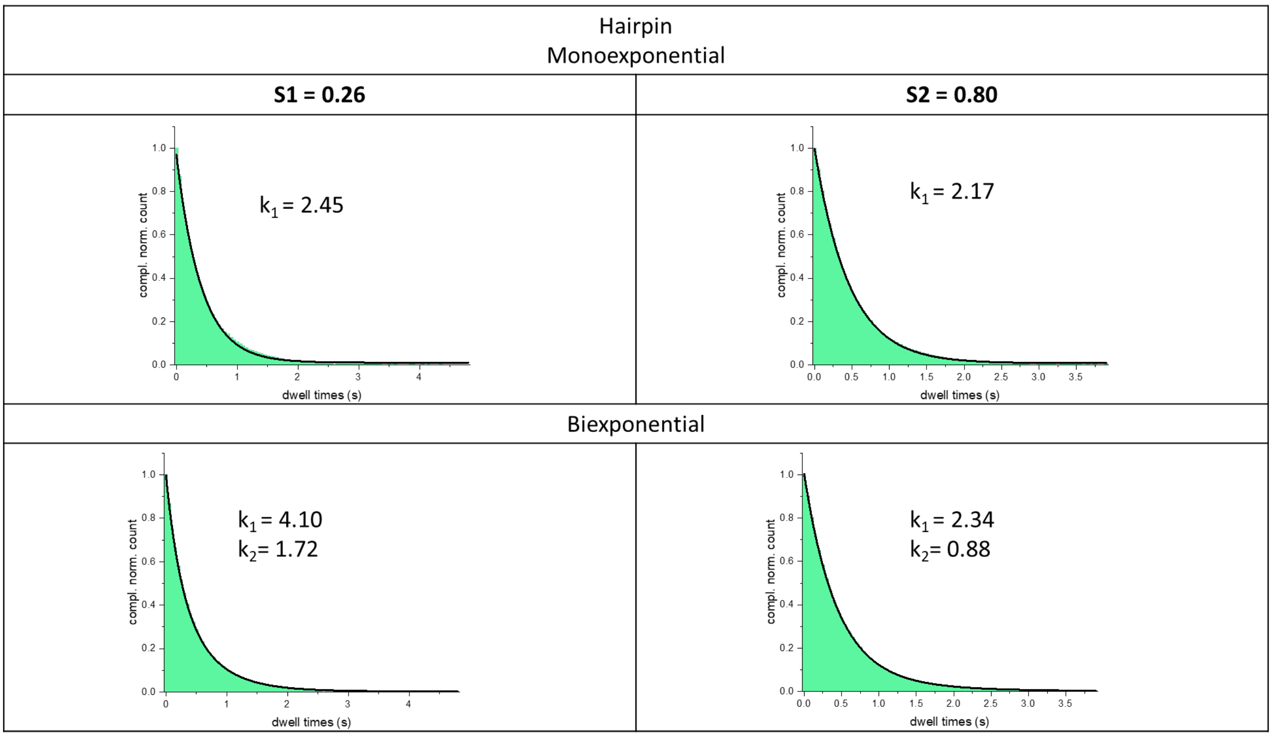

Figure S6. Cumulative frequency plots for DNA hairpin showing two-state dynamics.

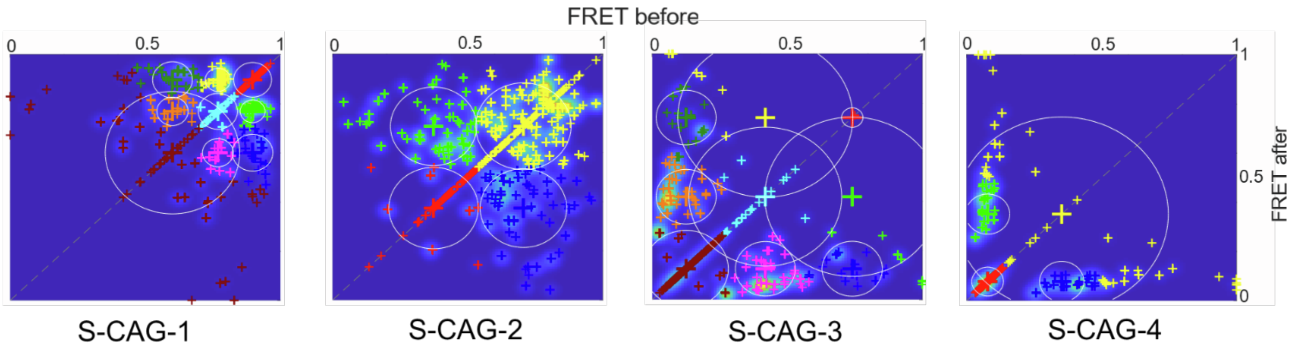

Figure S7. TDP from the unstitched trajectories of the static 3WJs.

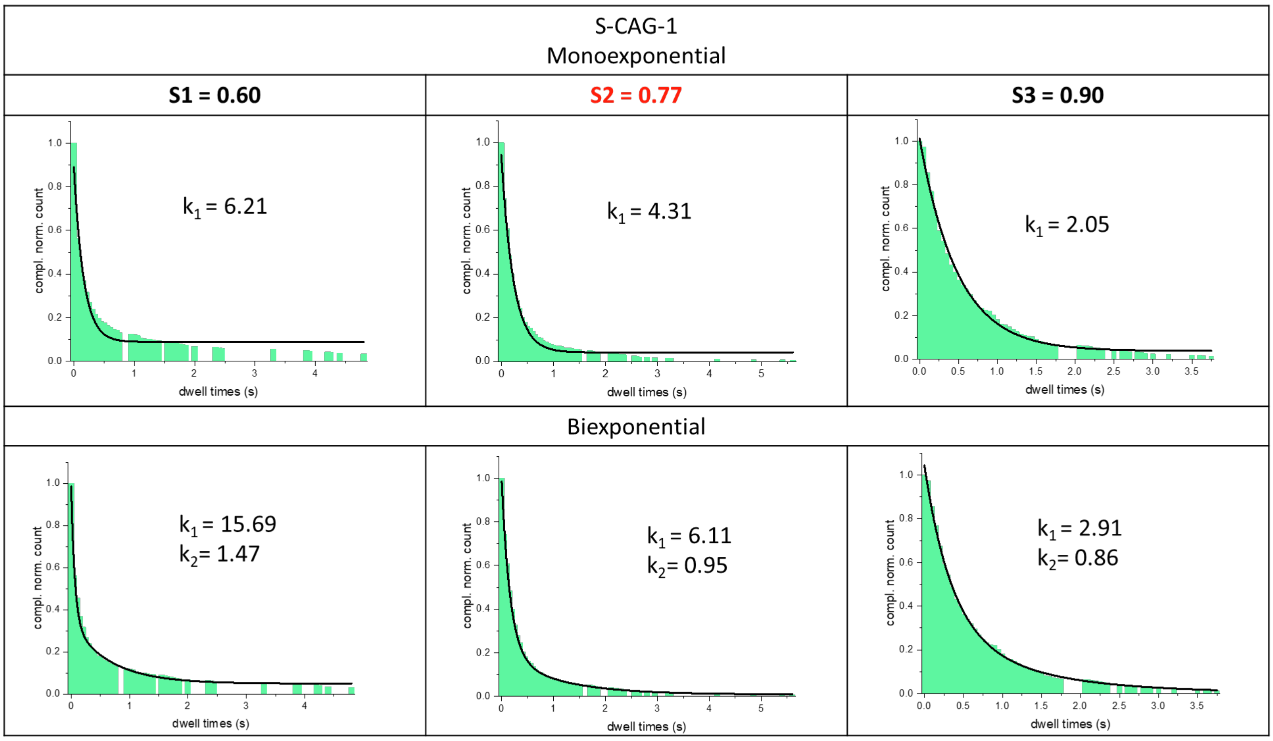

Figure S8. Cumulative frequency plots for S-CAG-1, a static $(CAG)_{10}$ 3WJ

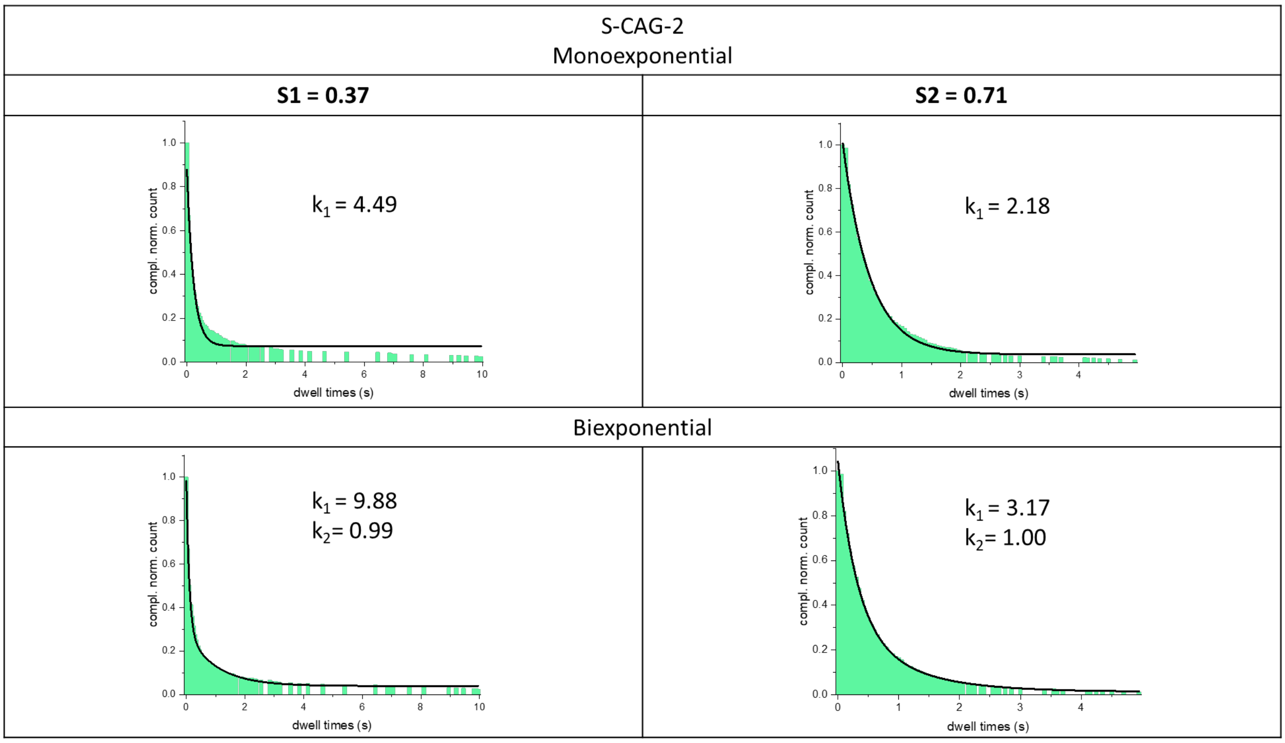

Figure S9. Cumulative frequency plots for S-CAG-2, a static $(CAG)_{10}$ 3WJ

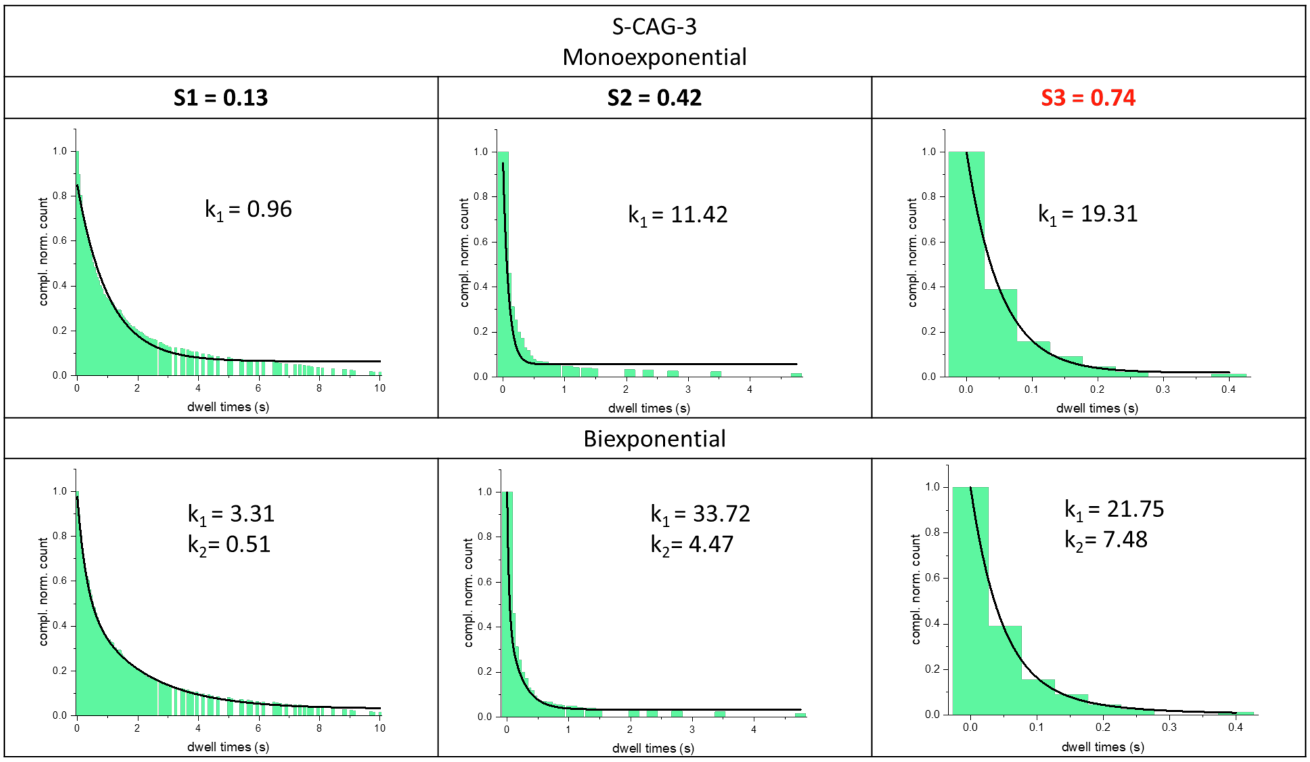

Figure S10. Cumulative frequency plots for S-CAG-3, a static $(CAG)_{10}$ 3WJ

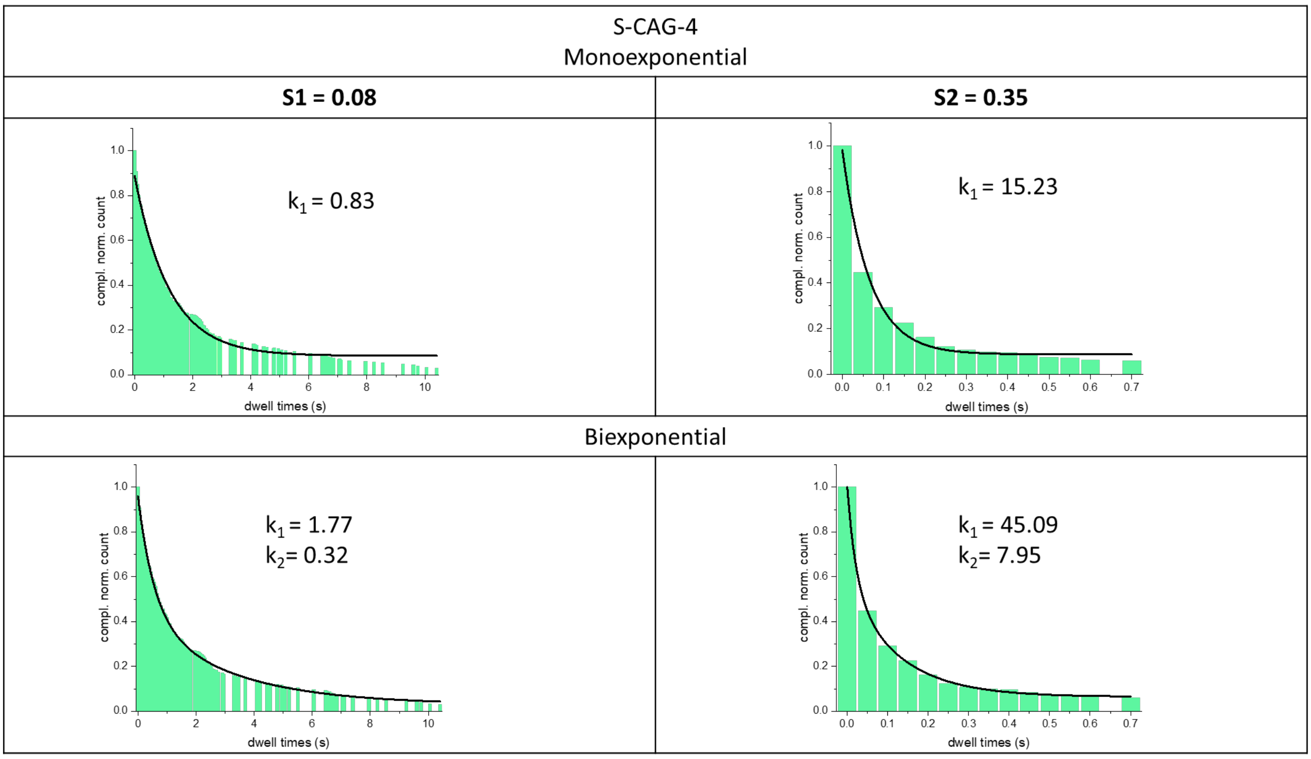

Figure S11. Cumulative frequency plots for S-CAG-4, a static $(CAG)_{10}$ 3WJ

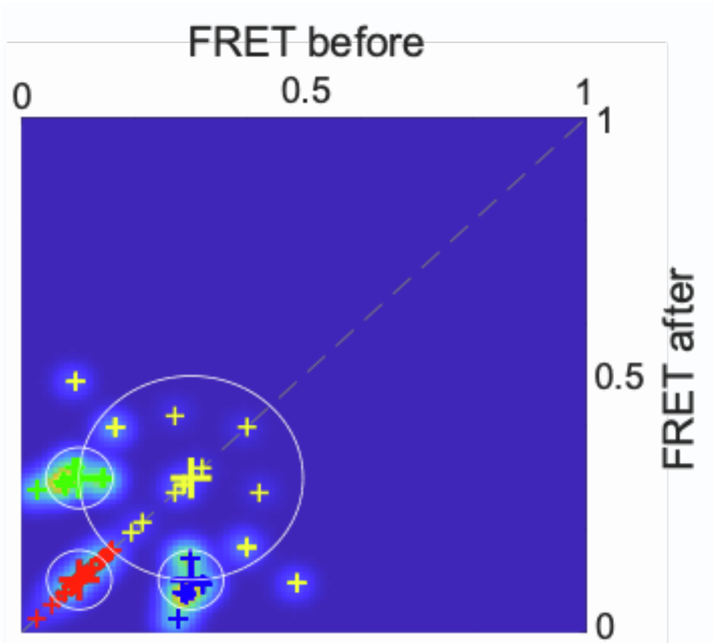

Figure S12. TDP from the unstitched trajectories of the fully complementary 3WJ showing two-state dynamics.

<table>
<tr><td colspan="2">Control: Fully Complementary 3WJ
Monoexponential</td></tr>
<tr><td>S1 = 0.10</td><td>S2 = 0.30</td></tr>
<tr><td>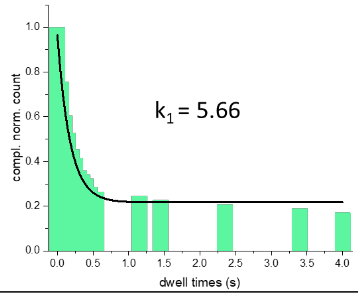
</td><td>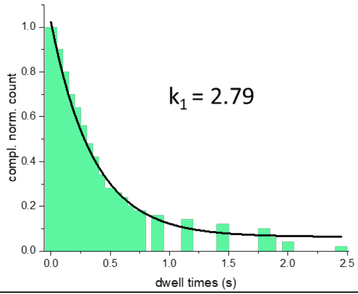
</td></tr>
<tr><td colspan="2">Biexponential</td></tr>
<tr><td>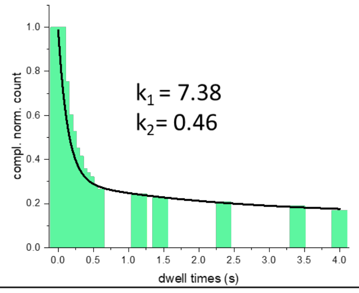
</td><td>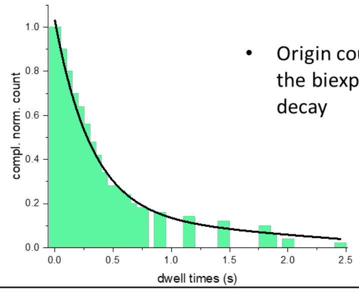

• Origin could not fit the biexponential decay</td></tr>
</table>

Figure S13. Cumulative frequency plots for a fully complementary 3WJ

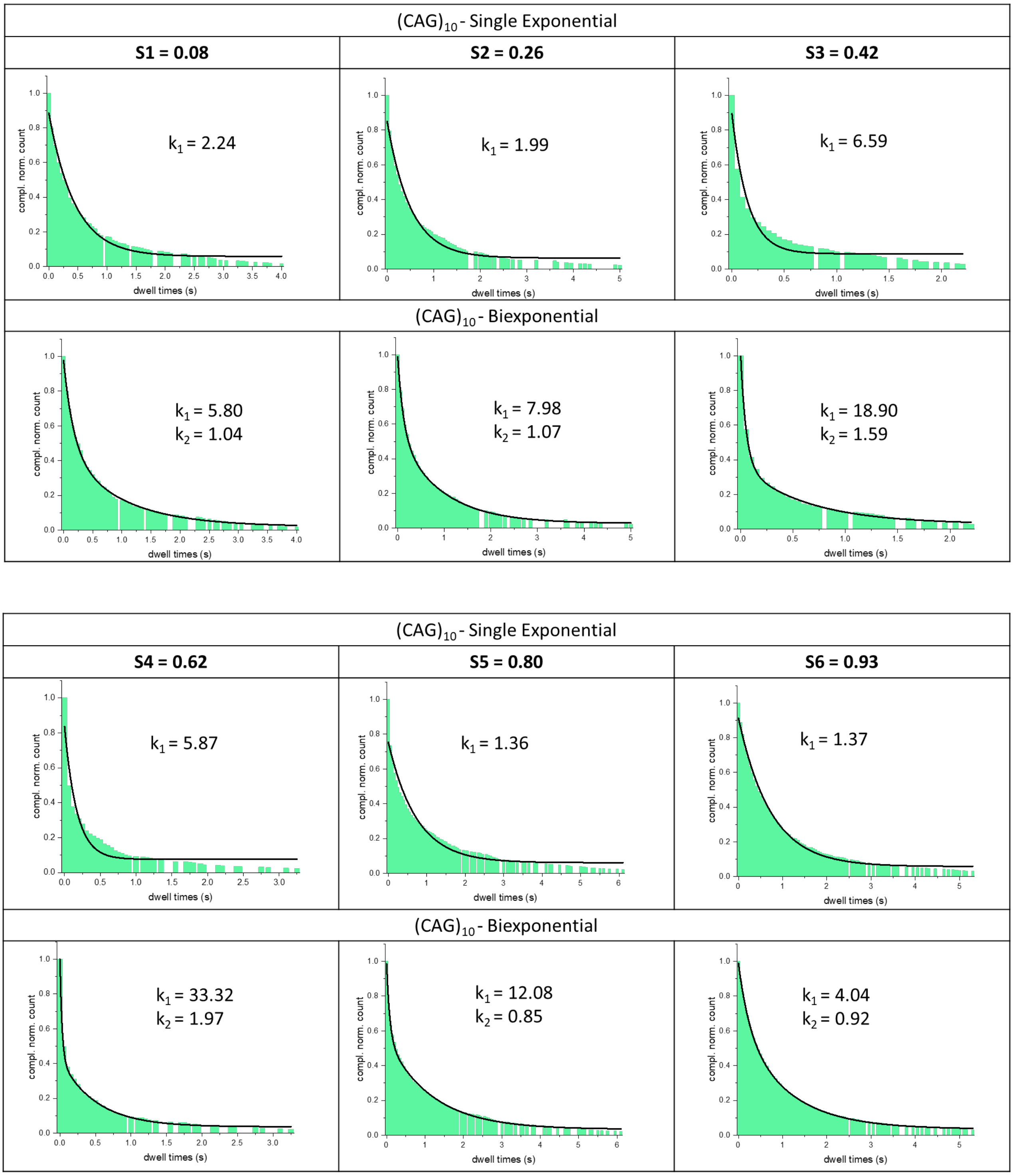

Figure S14. Cumulative frequency plots for mobile $(CAG)_{10}$

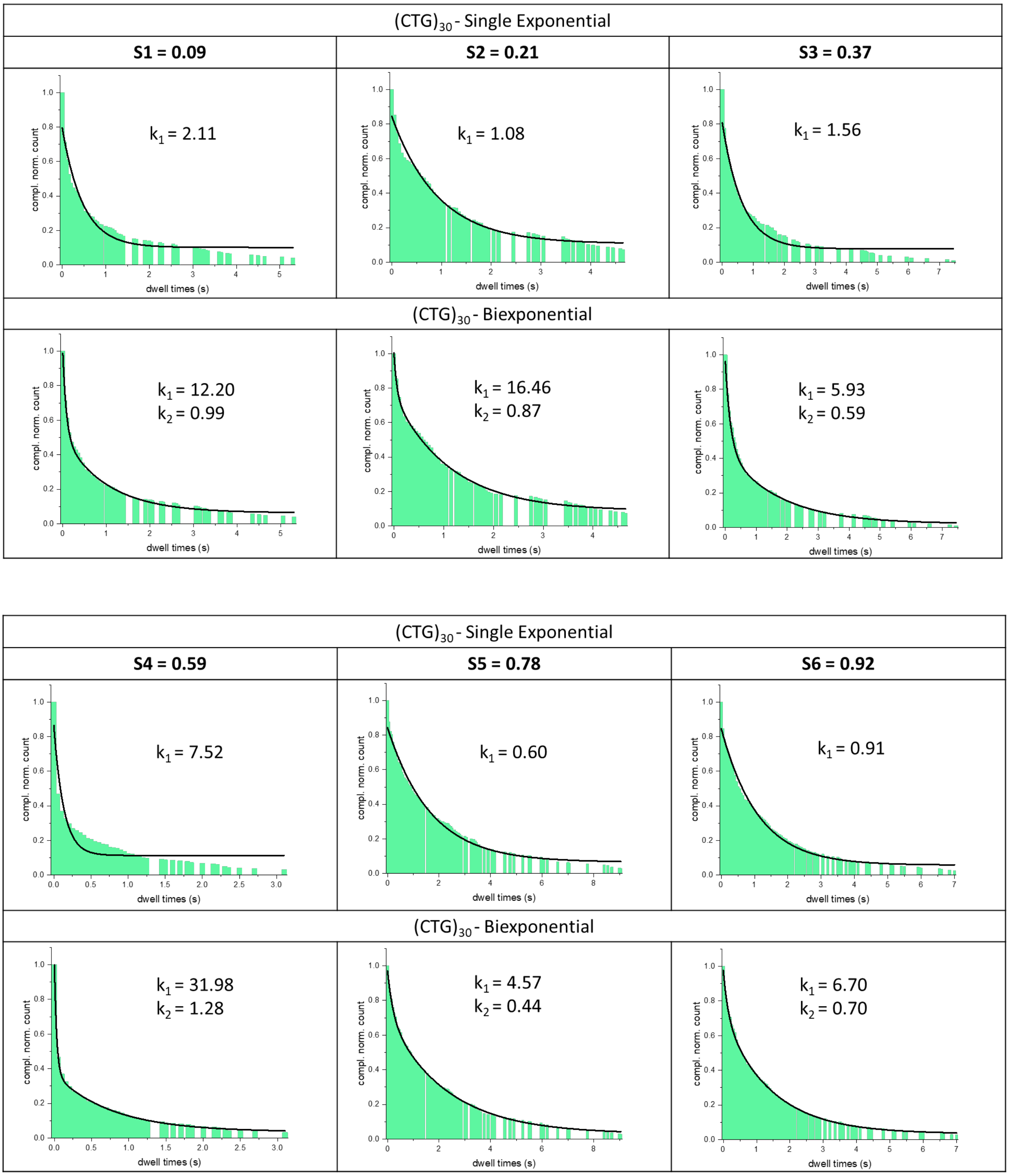

Figure S15. Cumulative frequency plots for mobile $(CTG)_{30}$

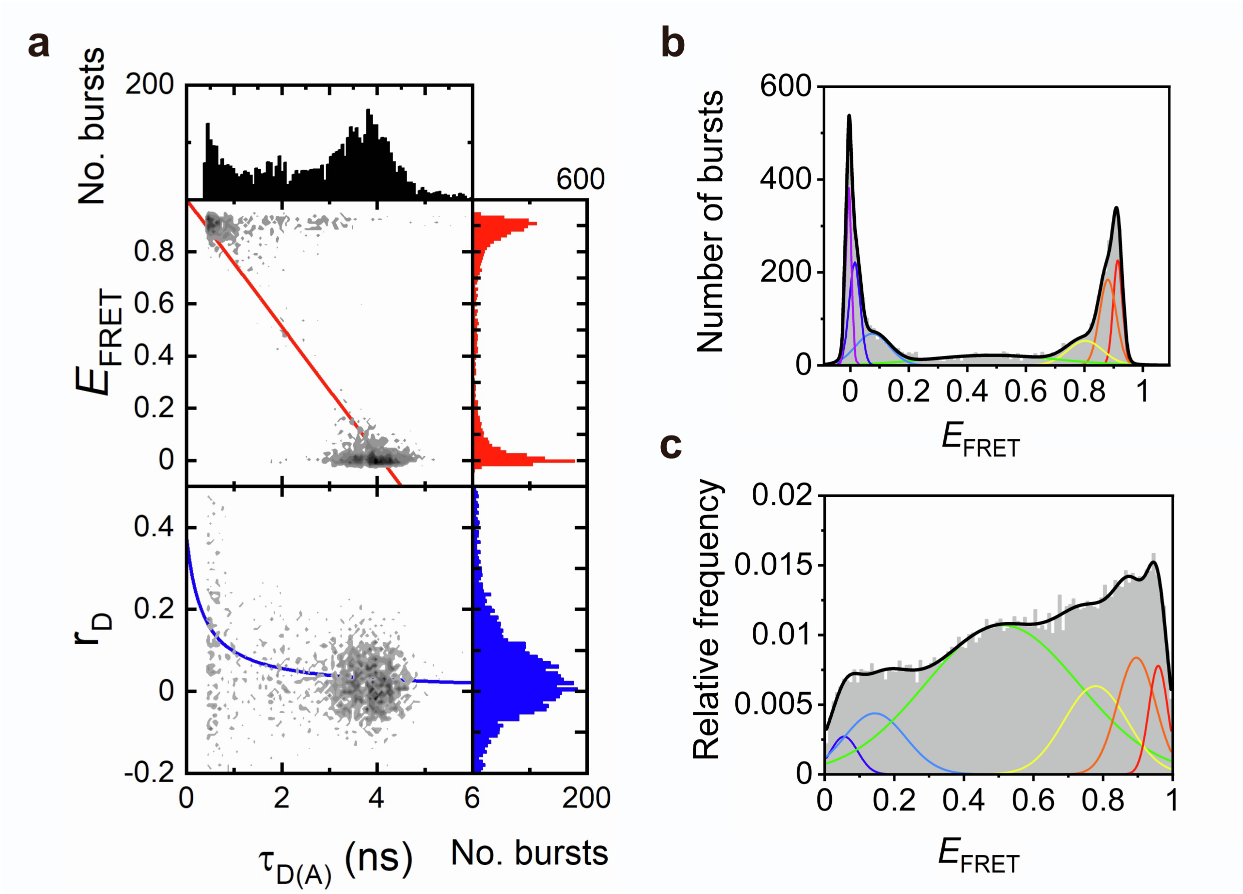

Figure S16. SM-FRET of mobile $(CAG)_{40}$ using MFD and TIRF. a) 2D MFD plot shows FRET efficiency (E_{FRET}) or donor anisotropy (r_D) vs donor lifetime [$\tau_{D(A)}$]. The overlaid red line is the theoretical FRET relationship $E = 1 - [\tau_{D(A)}/\tau_D]$, where τ_D = 4.1 ns. The blue overlaid line is the Perrin equation $r_D = r_0/[1 + \tau_{D(A)}/\rho_D]$, with mean rotational correlation time ρ_D = 0.35 ns and fundamental anisotropy r_0 = 0.375. The gray scale indicates an increasing number of single-molecule bursts from white to black. The sample was diluted into buffer containing 20 mM Tris, 15 mM NaCl, and 1 mM ascorbic acid at pH 7.5. Before the addition of sample, the buffer was stirred and cleaned with activated charcoal to remove fluorescent impurities. See Hu *et al.* (reference 19 in the main text) for further details of the MFD experiment. b) FRET histogram from MFD fitted to one donor-only state and six FRET states. c) FRET histogram from TIRF fitted to six FRET states using HaMMy.

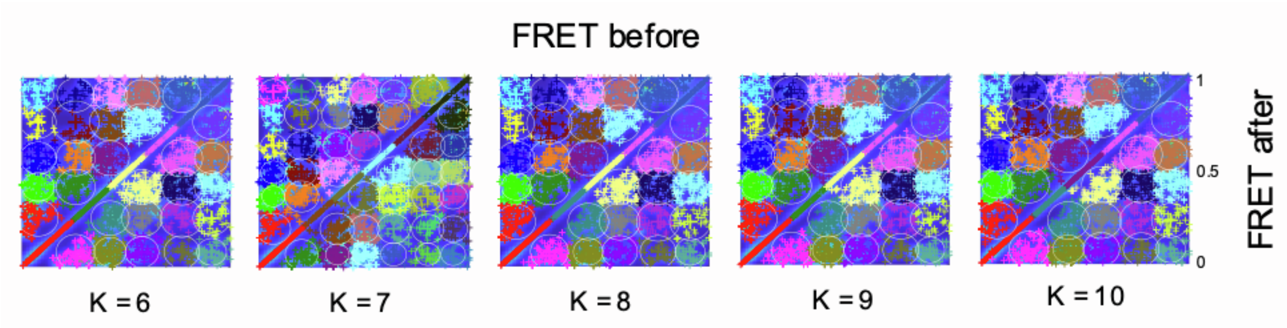

Figure S17. TDPs built from the unstitched trajectories of mobile $(CAG)_{40}$ for *K* = 6, 7, 8, 9, 10.

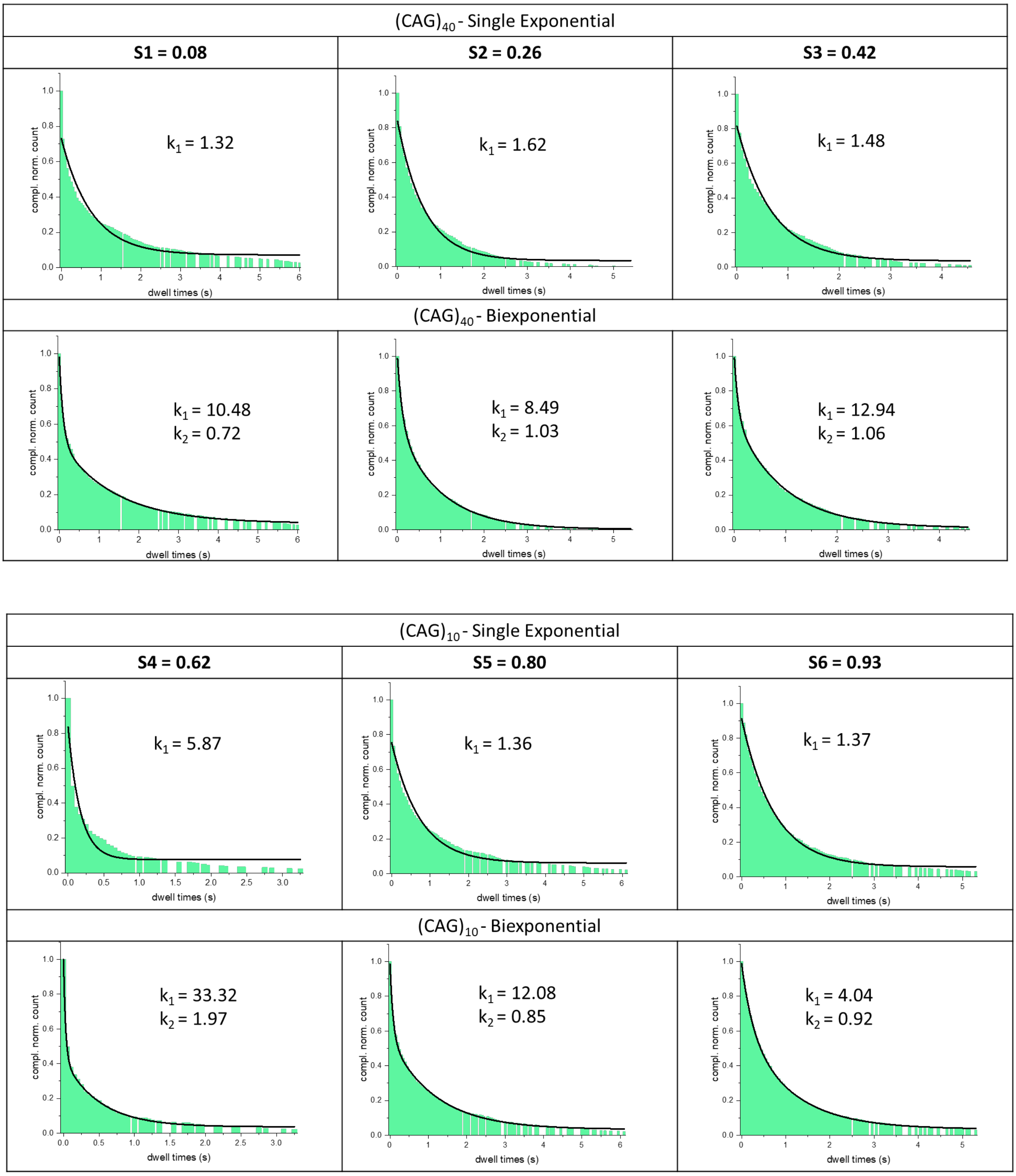

Figure S18. Cumulative frequency plots for mobile $(CAG)_{40}$
